# Thrombolytic therapy for patients with acute ischemic stroke: systematic review and network meta-analysis of randomized trials

**DOI:** 10.3389/fneur.2024.1490476

**Published:** 2025-01-07

**Authors:** Li-Chao-Yue Sun, Wen-Shu Li, Wei Chen, Zhao Ren, Chun-Xing Li, Ze Jiang, Le Wang, De-Li Wang, Qing Xie

**Affiliations:** ^1^Department of Pharmacy, Aerospace Center Hospital, Beijing, China; ^2^Department of Pharmacy, Beijing Shijitan Hospital, Capital Medical University, Beijing, China; ^3^Department of Pharmacy, Emergency General Hospital, Beijing, China; ^4^Department of Pharmaceutical, Beijing Tongren Hospital, Capital Medical University, Beijing, China; ^5^Department of Pharmaceutical, Sichuan Taikang Hospital, Chengdu, Sichuan, China; ^6^Department of Pharmacy, Maternal and Child Health Hospital of Guangxi Zhuang Autonomous Region, Nanning, Guangxi, China

**Keywords:** acute ischemic stroke, alteplase, tenecteplase, reteplase, network meta-analysis

## Abstract

**Objective:**

To systematically compare the benefits and risks of all thrombolytic agents (tenecteplase, reteplase, and alteplase) at different doses for thrombolytic therapy in patients with acute ischemic stroke (AIS).

**Background:**

Alteplase is the cornerstone treatment for AIS, but alternative thrombolytic agents are needed. The efficacy and safety of tenecteplase and reteplase, compared to alteplase, remain unclear, as does the optimal dosing for these treatments.

**Method:**

A systematic search was conducted in PubMed, Web of Science, SCOPUS, and the Cochrane Central Register of Controlled Trials (CENTRAL) for relevant English-language studies up to July 5, 2024. Randomized controlled trials (RCTs) comparing standard-dose alteplase with varying doses of tenecteplase or reteplase in AIS patients were included. Primary outcomes were functional outcome at 90 days, symptomatic intracranial hemorrhage, death within 90 days, and serious adverse events. Data on study characteristics, patient demographics, interventions, and outcomes were extracted, and bias risk assessed. A multivariate random-effects model was used for network meta-analysis to derive odds ratios (OR) and 95% confidence intervals (CI).

**Result:**

Twelve RCTs were included (10 with tenecteplase, 2 with reteplase) involving 6,633 patients, all compared against 0.9 mg/kg alteplase. In comparison with alteplase, tenecteplase demonstrated OR of 1.08 for achieving an excellent functional outcome at 90 days (95% CI: 0.97 to 1.22, *P* = 0.17). Reteplase, on the other hand, showed a significantly higher OR of 1.55 for the same outcome (95% CI: 1.23 to 1.95, *P* = 0.0002). Reteplase at 18 mg + 18 mg (OR 1.6, 95% CI: 0.91–2.5) showed a higher probability of achieving an excellent functional outcome at 90 days compared to alteplase. When considering a good functional outcome at 90 days, tenecteplase had an OR of 1.03 (95% CI: 0.81 to 1.3, *P* = 0.82), while reteplase had an OR of 1.15 (95% CI: 0.61 to 2.19, *P* = 0.66). Tenecteplase at 0.25 mg/kg (OR 1.3, 95% CI: 0.79–2.5) had the highest probability of achieving a good functional outcome at 90 days. For safety outcomes, 0.25 mg/kg tenecteplase had lower incidences of symptomatic intracranial hemorrhage (OR 0.88, 95% CI: 0.35–1.8), death within 90 days (OR 0.91, 95% CI: 0.54–1.4), and serious adverse events (OR 1.0, 95% CI: 0.47–2.3) compared to alteplase, though differences were not statistically significant. Reteplase at 18 mg + 18 mg had higher incidences of death within 90 days (OR 1.2, 95% CI: 0.48–3) and serious adverse events (OR 1.4, 95% CI: 0.4–5.0) compared to alteplase, without significant differences. Subgroup analysis showed better efficacy with 0.25 mg/kg tenecteplase in Asians (OR 1.18, 95% CI 0.96–1.45, *P* = 0.12) than in Caucasians (OR 1.08, 95% CI 0.9–1.3, *P* = 0.39).

**Conclusion:**

This study suggests that tenecteplase and reteplase are viable alternatives to alteplase for thrombolysis in AIS. Tenecteplase at 0.25 mg/kg and reteplase at 18 mg + 18 mg may offer better efficacy compared to standard-dose alteplase, although the risk of adverse events with reteplase should be considered. Tenecteplase at 0.25 mg/kg appears to provide the best benefit-risk profile based on current evidence. Further head-to-head trials of tenecteplase and reteplase are needed to determine the optimal thrombolytic agent and dosing.

**Systematic review registration:**

https://www.crd.york.ac.uk/prospero/, PROSPERO CRD42024566146.

## 1 Introduction

AIS is among the most common and life-threatening cerebrovascular diseases worldwide. Intravenous thrombolysis with alteplase within 4.5 h of symptom onset is the globally recognized cornerstone of AIS treatment. However, due to its short half-life, alteplase requires continuous infusion, increasing the complexity of patient care and limiting its clinical use (1–3). Tenecteplase, a genetically modified version of alteplase with a longer half-life, can be administered as a single bolus injection, offering similar clinical benefits to alteplase and has been frequently recommended by the European Stroke Organization (ESO) guidelines (4, 5). Similarly, reteplase, a recombinant plasminogen activator given in a double-bolus regimen (two injections 30 min apart with a fixed dose), has shown a higher likelihood of achieving excellent functional outcomes compared to alteplase (6, 7). However, due to a lack of direct comparative evidence, the relative advantages of alteplase, tenecteplase, and reteplase for intravenous thrombolysis in AIS patients remain unclear.

Previous meta-analyses on thrombolytic therapy for AIS have yielded conflicting results, often limited by the lack of high-quality data from randomized trials (8, 9). This study addresses these limitations by exclusively including RCTs and overcoming other constraints: (1) We expanded the outcome measures to include death within 90 days and serious adverse events as safety indicators. (2) We conducted a network meta-analysis of different doses of tenecteplase and reteplase. (3) We performed subgroup analyses based on race and age.

The objectives of this systematic review and meta-analysis are: (1) To assess the efficacy and safety of alteplase, tenecteplase, and reteplase in the treatment of AIS. (2) To determine the optimal doses of tenecteplase and reteplase for AIS treatment. (3) To explore the impact of race and age on intravenous thrombolysis outcomes in AIS patients.

## 2 Method

### 2.1 Registration

This review follows the pre-specified protocol registered with PROSPERO (CRD42024566146). Differences between this review and the original PROSPERO protocol are detailed in Supplementary Table S1. This report adheres to the Preferred Reporting Items for Systematic Reviews and Meta-Analyses (PRISMA) guidelines for network meta-analyses (10). Ethical approval was not required as this study was primarily analyzed using data from existing RCTS in the database.

### 2.2 Eligibility criteria

Inclusion criteria for this network meta-analysis:

1. Studies utilizing all thrombolytic drugs for intravenous thrombolysis.
2. Large-scale phase 2/3 RCTs.
3. Studies involving adult patients (aged 18 years and above) undergoing intravenous thrombolysis who meet the standard criteria for thrombolysis.
4. Studies reporting at least one outcome measure of interest for this meta-analysis.

Exclusion criteria for this network meta-analysis:

1. Studies combining antiplatelet therapy with thrombolysis.
2. Studies involving mechanical thrombectomy.
3. Studies not published in English.
4. Studies classified as fundamental experimental research, conference abstracts, case reports, or reviews.
5. Studies lacking a comparison group.
6. Studies presenting overlapping participant data.

### 2.3 Outcomes

The interventions of interest included different doses of tenecteplase (0.1 mg/kg, 0.25 mg/kg, 0.4 mg/kg) and reteplase (12 mg+12 mg, 18 mg+18 mg), compared to the standard dose of alteplase (0.9 mg/kg). Studies comparing these interventions against each other or against alteplase were included. Exclusion criteria are detailed in the Supplementary Table S2. Primary outcomes included functional outcomes at 90 days, determined by the modified Rankin Scale (mRS), including excellent functional outcome (mRS 0-1, or no change from baseline) and good functional outcome (mRS 0-2, or no change from baseline). Safety outcomes included symptomatic intracranial hemorrhage (sICH), death within 90 days, and serious adverse events (SAEs). Additional outcomes such as any parenchymal hemorrhage, any intracranial hemorrhage (ICH), asymptomatic ICH, and major neurological improvement within 72 h were initially considered but ultimately excluded due to insufficient data.

### 2.4 Data sources and searches

Two authors (Li-chao-yue Sun and Wen-shu Li) conducted a comprehensive search of PubMed, Web of Science, SCOPUS, and the Cochrane CENTRAL for relevant English-language studies up to July 2024. The search strategy included terms such as “stroke,” “tenecteplase,” “reteplase,” “alteplase,” and “randomized.” Additional eligible trials were identified from two published systematic reviews (11, 12). Detailed search strategies are provided in the Supplementary Table S3.

### 2.5 Data extraction and quality assessment

Two authors independently screened titles, abstracts, and full texts for eligibility, with discrepancies resolved by a third reviewer (Li-chao-yue Sun, Wen-shu Li, Wei Chen). Four researchers (Ze Jiang, Li-chao-yue Sun, Wen-shu Li, Wei Chen) independently extracted data using standardized forms, including study characteristics, patient demographics, intervention details, and outcomes of interest. Any disagreements were resolved through consensus with a third evaluator. The risk of bias for eligible RCTs was independently assessed using the Cochrane Collaboration's risk of bias tool (13). Each study was evaluated for low, unclear, or high risk of bias across multiple domains.

### 2.6 Data synthesis and analysis

For each outcome, we first conducted pairwise meta-analyses using fixed/random effects models to estimate pooled OR and 95% CI. Heterogeneity was assessed using the I^2^ statistic. For efficacy and safety outcomes, we ranked the probabilities of each treatment (alteplase, two doses of reteplase, and three doses of tenecteplase) using surface under the cumulative ranking (SUCRA) curves. Data analysis and bias assessments were performed using Review Manager Version 5.3, Stata 16 (mvmeta command and network routine), and the “rjags” and “gemtc” packages in R software (version 4.4) (14).

### 2.7 Sensitivity and subgroup analyses

The Supplementary material describes the methods for assessing consistency and publication bias (funnel plots and Egger's regression test). We examined potential sources of heterogeneity, including geographic regions (Caucasians and Asians), mean age differences (dichotomized at 65 years), baseline National Institutes of Health Stroke Scale (NIHSS) scores (0–5: low/minor stroke, 6–15: moderate, 15–20: moderate-high, >21: high), and gender. Subgroup analyses were performed for overall tenecteplase, reteplase, and alteplase (regardless of dose), as well as specific doses of tenecteplase (0.25 mg/kg) and reteplase (18 mg+18 mg) compared to the standard dose of alteplase.

## 3 Result

### 3.1 Systematic review and characteristics

**Figure 1:**
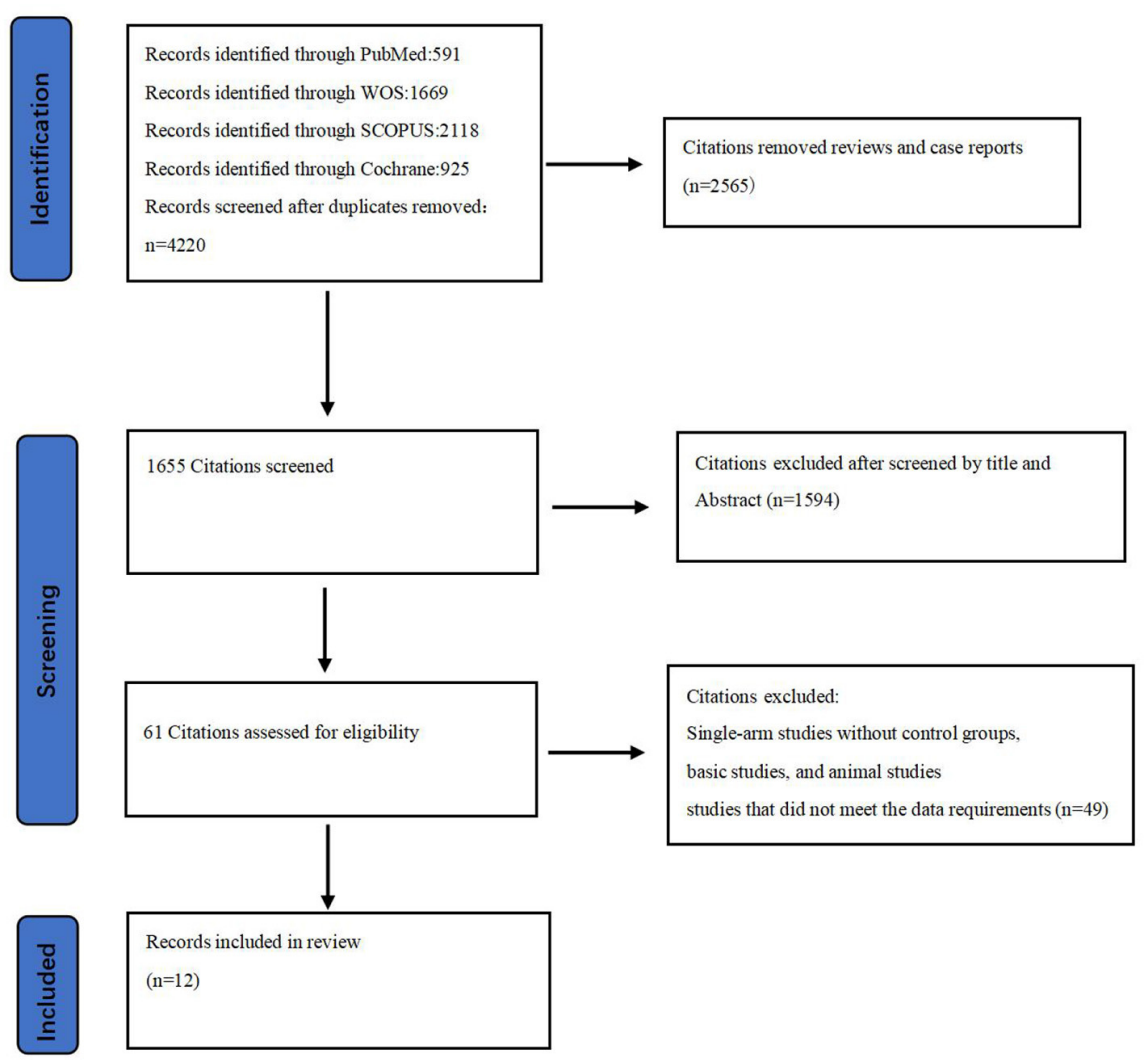
Study flow diagram for evidence source and selection.

Among the 4,220 non-duplicate studies screened, 12 RCTs (involving 6,633 acute stroke patients) met the inclusion criteria for this study (Figure 1) (15–26). These RCTs provided at least one outcome included in our network meta-analysis. Detailed reasons for exclusion are available in the Supplementary material, and Table 1 documents the basic characteristics and outcomes of the included RCTs.

**Table 1:** The characteristics of included RCTs.

**References**	**Recruitment time**	**Country**	**Publication date**	**RCT number**	**Intervention**	**No. patients**	**Age, mean (SD)**	**Male sex (%)**	**Time of onset to treatment**	**Baseline NIHSS**	**Outcomes**
Haley et al. (15)	2006–2008	United States	2010	NA	TNK (0.1/0.25/0.4 mg/kg)	31:31:19:31	TNK0.1:67 ± 16; TNK0.25:69 ± 15; TNK0.4:68 ± 16; rt–PA:72 ± 16	TNK0.1: 39; TNK0.25: 52; TNK0.4: 68; rt-PA:16	NA	TNK 0.1: 8 (5–11); TNK 0.25: 10 (6–15); TNK 0.4: 9; (5–17) rt-PA: 13 (5–17)	mRS at 90 days, sICH, death within 90 days,
Parsons et al. (22)	2008–2011	Australia	2012	ACTRN12608000466347	TNK (0.1/0.25mg/kg)	25:25:25	TNK0.1:72 ± 6.9; TNK0.25:68 ± 9.4; rt–PA:70 ± 8.4	TNK0.1:52; TNK0.25:52; rt-PA:48	TNK0.1:3.1 ± 0.9; TNK0.25:3.0 ± 0.7; rt-PA:12.7 ± 0.8	TNK0.1:14.5 ± 2.3 TNK0.25:14.6 ± 2.3; rt-PA:14 ± 2.3	mRS at 90 days, sICH, death within 90 days,
ATTEST; Huang et al. (18)	2012–2013	Scotland	2015	NCT01472926	TNK (0.25mg/kg)	47:49	TNK0.25:71 ± 13; rt–PA:71 ± 12	TNK0.25:64; rt-PA:63	TNK0.25:184 ± 44; rt-PA:192 ± 54	TNK0.25: 12 (9–18); rt-PA: 11(8–16)	mRS at 90 days, sICH, death within 90 days, SAE
NOR-TEST; Logallo et al. (20)	2012–2016	Norway	2017	NCT01949948	TNK (0.4mg/kg)	549:541	TNK0.4:70.8 ± 14.4; rt–PA:71.2 ± 13.2	TNK0.4:58; rt-PA:62	TNK0.4:118(79-180); rt-PA: 111(80-174)	TNK0.4:4(2-7); rt-PA: 4(2-8)	mRS at 90 days, sICH, death within 90 days, SAE
Campbell et al. (17)	2015–2017	Australia and New Zealand	2018	NCT02388061	TNK (0.25mg/kg)	101:101	TNK0.25:70.4 ± 15.1; rt–PA:71.9 ± 13.7	TNK0.25:57; rt-PA:51	TNK0.25:125(105-156); rt-PA:134(104-176)	TNK0.25:17(12-22); rt-PA:17(12-22)	mRS at 90 days, sICH, death within 90 days, SAE
TRACE; Li et al. (19)	2018–2020	China	2022	NCT04676659	TNK (0.1/0.25/0.32 mg/kg)	60:57:60:59	TNK0.1:62.4 ± 11.1; TNK0.25:64.3 ± 12.8; TNK0.4:64.8 ± 12.1; rt–PA:66.5 ± 12.6	TNK 0.1: 80; TNK 0.25: 74; TNK 0.32: 70; rt-PA: 64	TNK 0.1: 154 (56–195); TNK 0.25: 149 (80–179); TNK 0.32: 147 (69–220); rt-PA: 153 (18–187)	TNK 0.1: 7.0 (5–10); TNK 0.25: 8 (5–12); TNK 0.32: 7.5 (6–12); rt-PA: 8 (5–12)	mRS at 90 days, sICH, death within 90 days, SAE
TASTE-A; Bivard et al. (16)	2019–2021	Australia	2022	NCT04071613	TNK (0.25mg/kg)	55:49	TNK0.25:76 (60–84) rt–PA:73(61–80)	TNK0.25:60; rt-PA:61	TNK0.25: 97 (68–157) rt-PA: 92 (66–31)	TNK: 8 (5–14); rt-PA: 8 (5–17)	mRS at 90 days, death within 90 days,
NOR-TEST2 (PARTA); Kvistad et al. (24)	2019–2021	Norway	2022	NCT03854500	TNK (0.4mg/kg)	96:101	TNK0.4: 73.2 ± 12.6; rt–PA: 68.6 ± 15.6	TNK0.4: 45; rt-PA: 51	TNK0.4: 92.5 (74–143); rt-PA: 99 (73–143)	TNK0.4: 11.5 (8–17); rt-PA: 11 (8–17.6)	mRS at 90 days, sICH, death within 90 days,
AcT; Menon et al. (21)	NA	Canada	2022	NCT03889249	TNK (0.25mg/kg)	806:711	TNK0.25:74 (63–83) ;rt–PA:73 (62–83)	TNK0.25:52.6;rt-PA:51.6	TNK0.25:128 (93–186) ;rt-PA:131 (95–188)	TNK0.25:9 (6–16) rt-PA:10 (6–17)	mRS at 90 days, sICH, death within 90 days, SAE
TRACE-2; Wang et al. (23)	2021–2022	China	2023	NCT04797013	TNK (0.25mg/kg)	705:696	TNK0.25:67 (58–73) rt–PA:65 (58–72)	TNK0.25:69; rt-PA:68	TNK0.25:180(135-222); rt-PA:178.5(135-230)	TNK0.25:7(5-10); rt-PA:7(6-10)	mRS at 90 days, sICH, death within 90 days, SAE
Li et al. (26)	2019–2021	China	2024	NCT04028518	Reteplase (18mg+18mg); Reteplase (12mg+12mg)	66:60:50	Ret12:62.8(10.1) Ret18:61.9(9.5) rt–PA:63.3(9.5)	Ret12:76.7 Ret18:69.7 rt-PA:76	Ret12:213.5 (162-241.5); Ret18:212.0 (163.0-235.0); rt-PA:215(162-244)	Ret12:6.0(5-8.5); Ret18:6(5-8); rt-PA:8(5-10)	mRS at 90 days, sICH, death within 90 days, SAE
RAISE; Li et al. (25)	2022–2023	China	2024	NCT05295173	Reteplase (18mg+18mg)	707:705	Ret18:63 (56–70); rt–PA:63 (56–70)	Ret18:71.9; rt-PA:69.2	Ret18:180 (131–221); rt-PA:183 (139–222)	Ret18:6(5-8); rt-PA:6(5-8)	mRS at 90 days, sICH, death within 90 days, SAE

1. RCT, randomized controlled trials; TNK, tenecteplase; ALT/rt-PA, alteplase; Ret, reteplase; sICH, symptomatic intracranial haemorrhage; SAE, serious adverse event.

TNK0.1: 0.1mg/kg tenecteplase, TNK0.25: 0.25mg/kg tenecteplase, TNK0.4: 0.4mg/kg tenecteplase, Ret12: 12mg+12mg reteplase, Ret18: 18mg+18mg reteplase.

2. Time of onset to treatment is usually measured in minutes/min.

**Figure 2:**
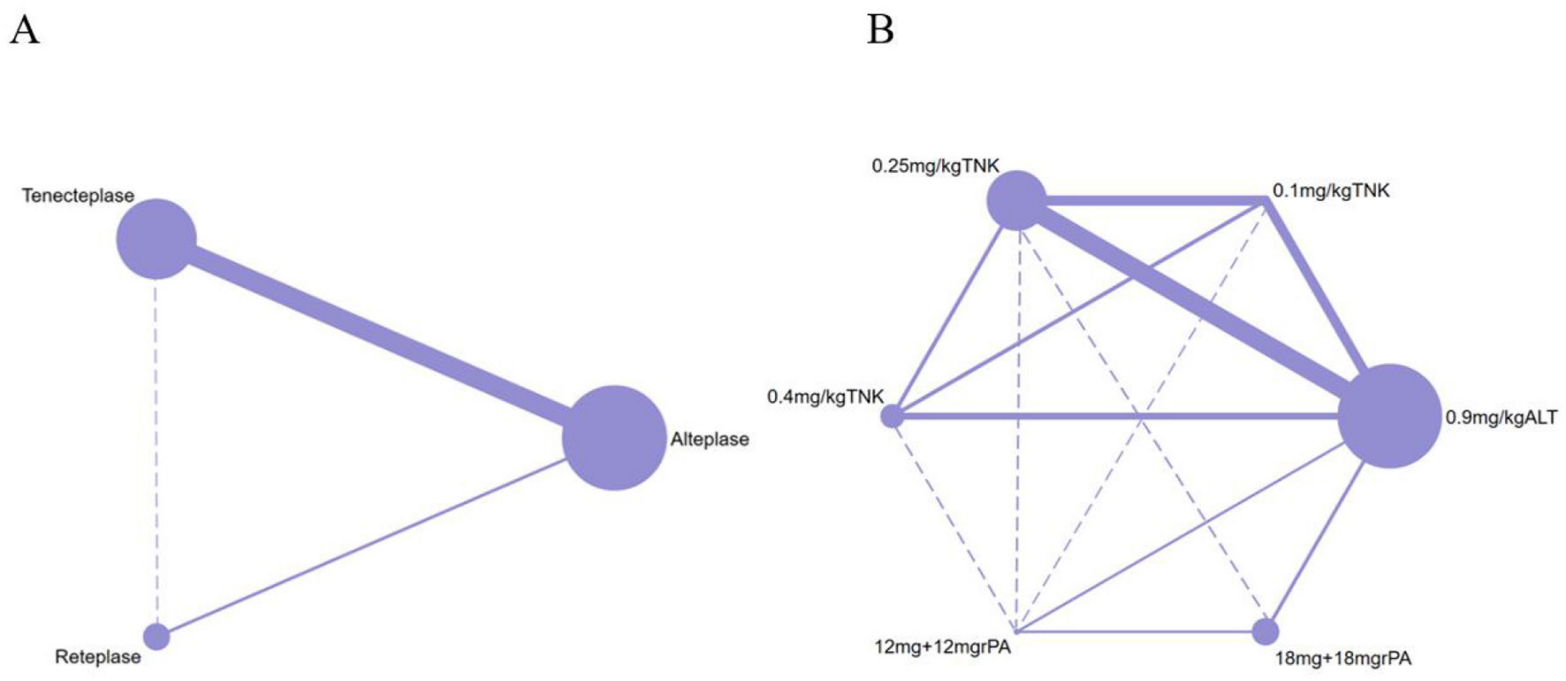
Network plot of studies included in network meta-analysis. **(A)** The network of included studies by type of thrombolytic drug. **(B)** The network of included studies by type and dose of thrombolytic drugs. ALT, Alteplase; TNK, Tenecteplase; rPA, Reteplase. Each node represents a treatment modality, with its size proportional to the number of patients receiving that treatment. The lines connecting two nodes indicate direct comparisons between the two treatment modalities, with the thickness of each line proportional to the number of trials comparing those two treatments. Dashed lines indicate that no direct comparison exists between the two thrombolytic treatments.

Of the 12 RCTs included in the network meta-analysis, 10 (83.33%) directly compared the efficacy and safety of tenecteplase with alteplase for treating acute stroke, while 2 compared reteplase with alteplase (25, 26). Based on dosage, 8 RCTs reported comparisons between 0.25 mg/kg tenecteplase and alteplase, 3 reported on 0.1 mg/kg tenecteplase, 3 on 0.4 mg/kg tenecteplase, 2 on 18 mg + 18 mg reteplase, and 1 on 12 mg + 12 mg reteplase. The outcome measures in RCTs exhibit variations in their definitions. In assessing excellent functional outcomes at 90 days, Campbell et al. (17), TASTE-A (16), and NOR-TEST2 (PARTA) (24) define it as a mRS score of 0–1 or a return to baseline, whereas nine other RCTs define it as an mRS score of 0–1 at 90 days. Regarding sICH, ATTEST (18), Campbell et al. (17), TASTE-A (16), and Li2024 (26) define it according to the SITS-MOST criteria; NOR-TEST (20), TRACE (19), NOR-TEST2 (PARTA) (24), AcT (21), TRACE-2 (23), and RAISE (25) define it based on the ECASS III criteria. For details on the definitions of efficacy and safety outcomes, refer to Supplementary Table S5. All RCTs employed a parallel control design with the control group receiving 0.9 mg/kg alteplase; 8 trials had two groups, 3 had three groups, and 1 had four groups (Figure 2). The network map for different outcomes is shown in Supplementary Figure S1.

### 3.2 Quality assessment

**Figure 3:**
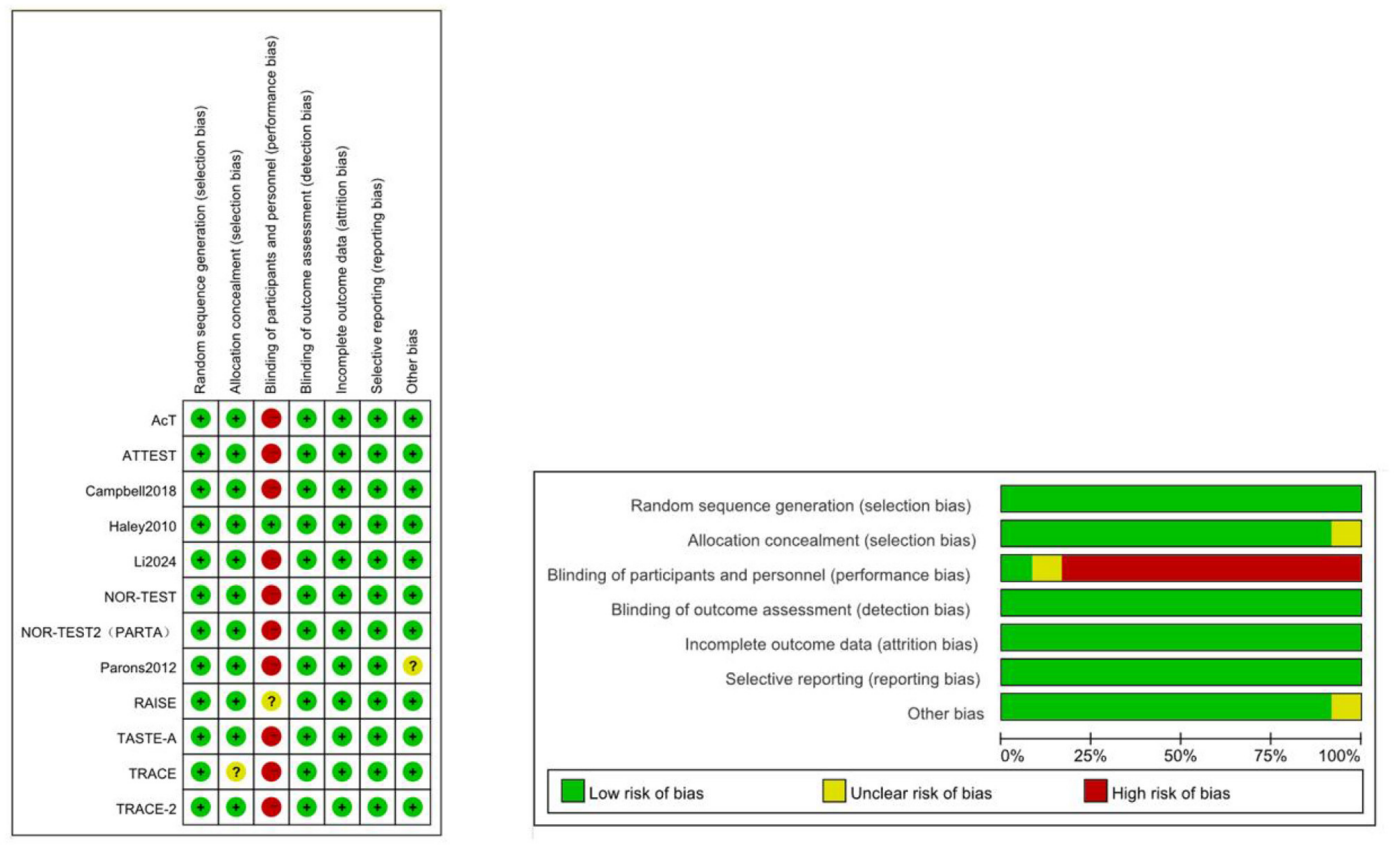
Cochrane risk of bias tool.

Due to the lack of blinding for participants and personnel, most studies were deemed to have a high risk of bias (Figure 3). Green represents low risk, yellow indicates unclear risk, and red denotes high risk. The direct comparisons between 0.25 mg/kg tenecteplase and 18 mg + 18 mg reteplase with alteplase contributed significantly to the network (Supplementary Figure S2). The RAISE trial did not provide blinding details, and the TRACE trial did not describe the allocation method, resulting in an unclear risk assessment.

### 3.3 Benefits

**Figure 4:**
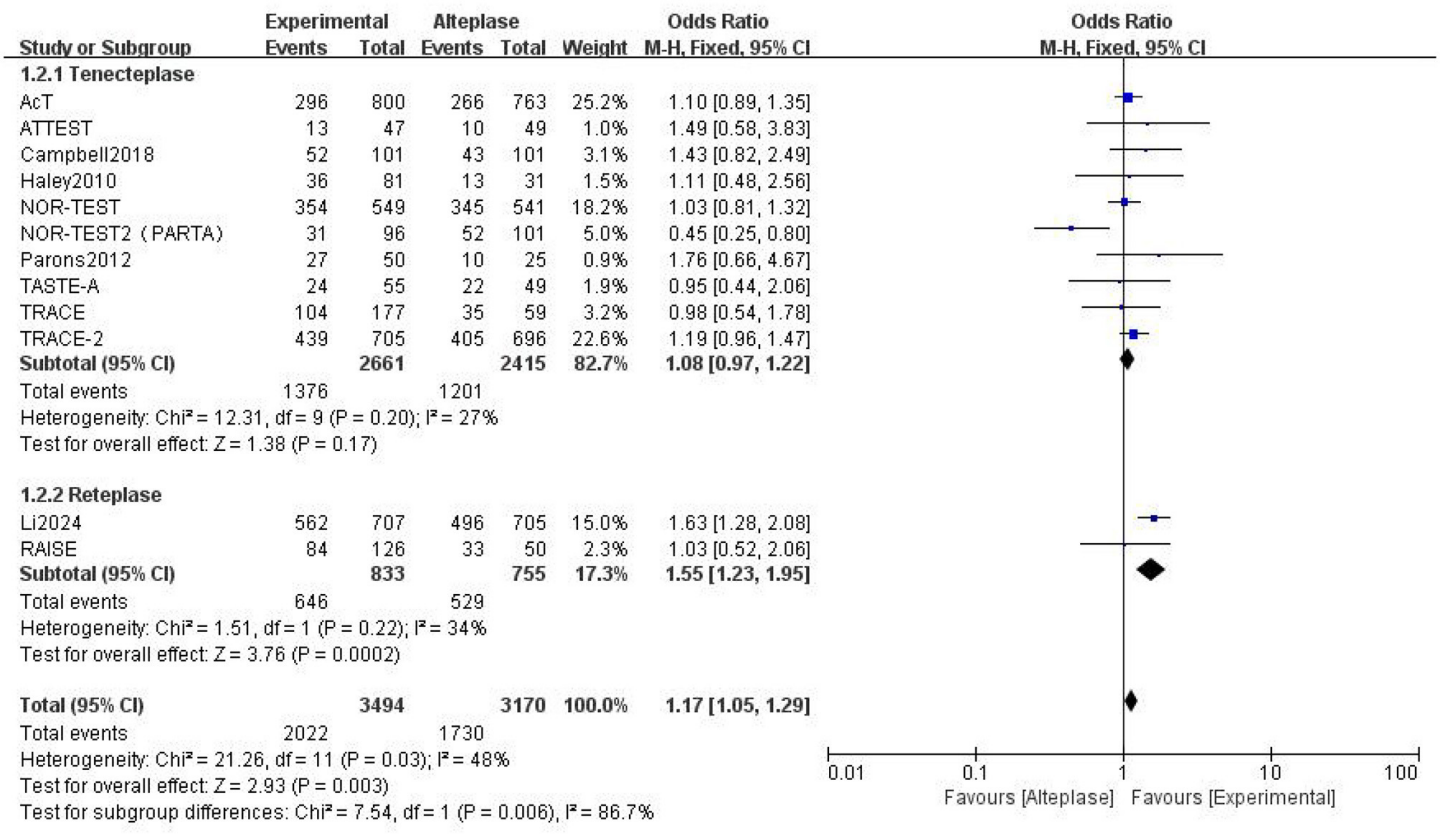
Forest plot for the tenecteplase and reteplase on excellent functional outcome at 90 days.

**Figure 5:**
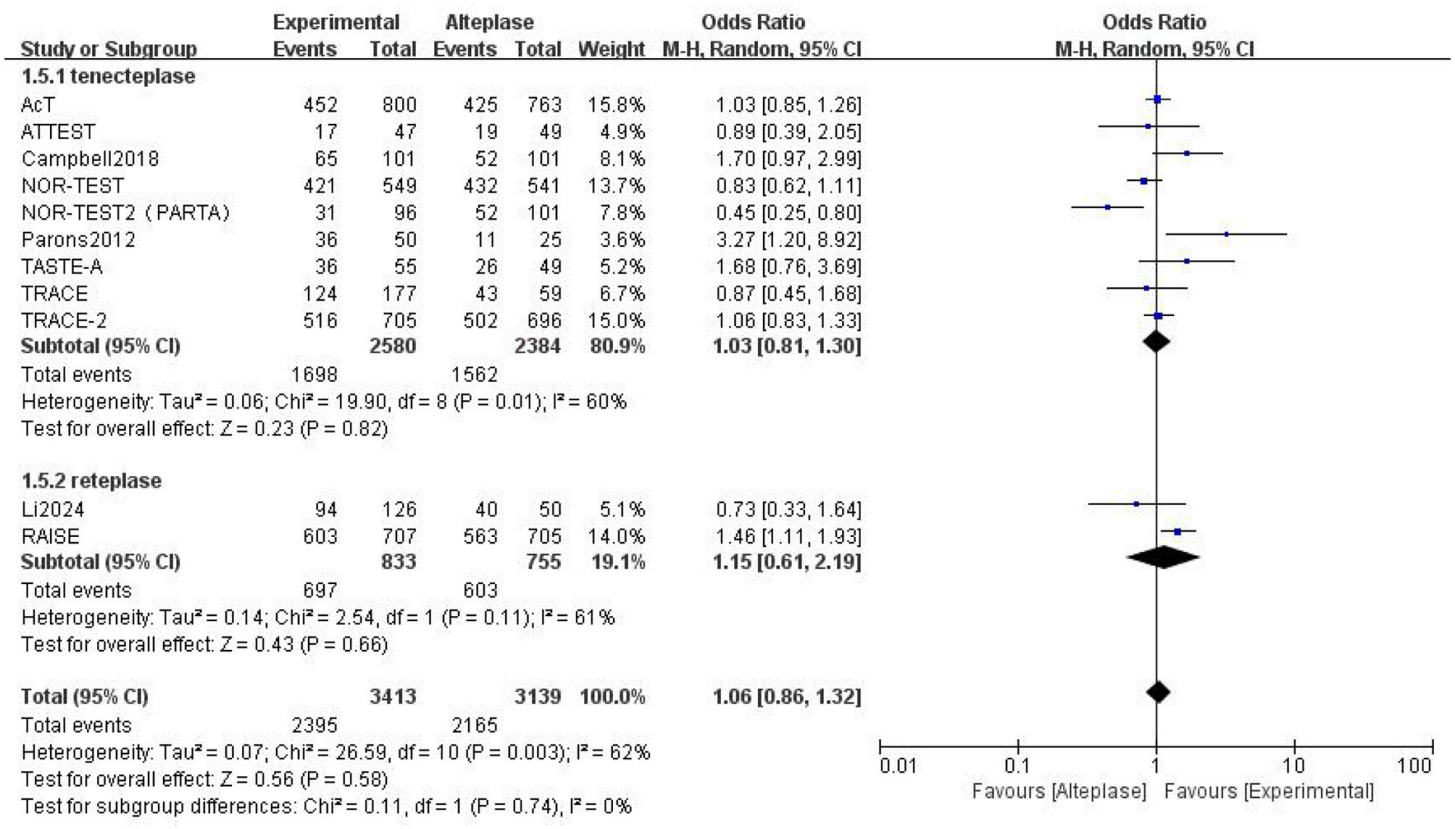
Forest plot for the tenecteplase and reteplase on good functional outcome at 90 days.

Compared to alteplase, tenecteplase showed no significant difference (excellent: OR 1.08, 95% CI: 0.97–1.22, I^2^ = 27%). However, patients treated with reteplase had better outcomes (excellent: OR 1.55, 95% CI: 1.23–1.95, I^2^ = 34%, *P* = 0.0002) (Figure 4). For good functional outcome at 90 days, neither tenecteplase (OR 1.03, 95% CI: 0.81–1.3, I^2^ = 60%) nor reteplase (OR 1.15, 95% CI: 0.61–2.19, I^2^ = 62%) showed significant differences compared to alteplase (Figure 5).

**Figure 6:**
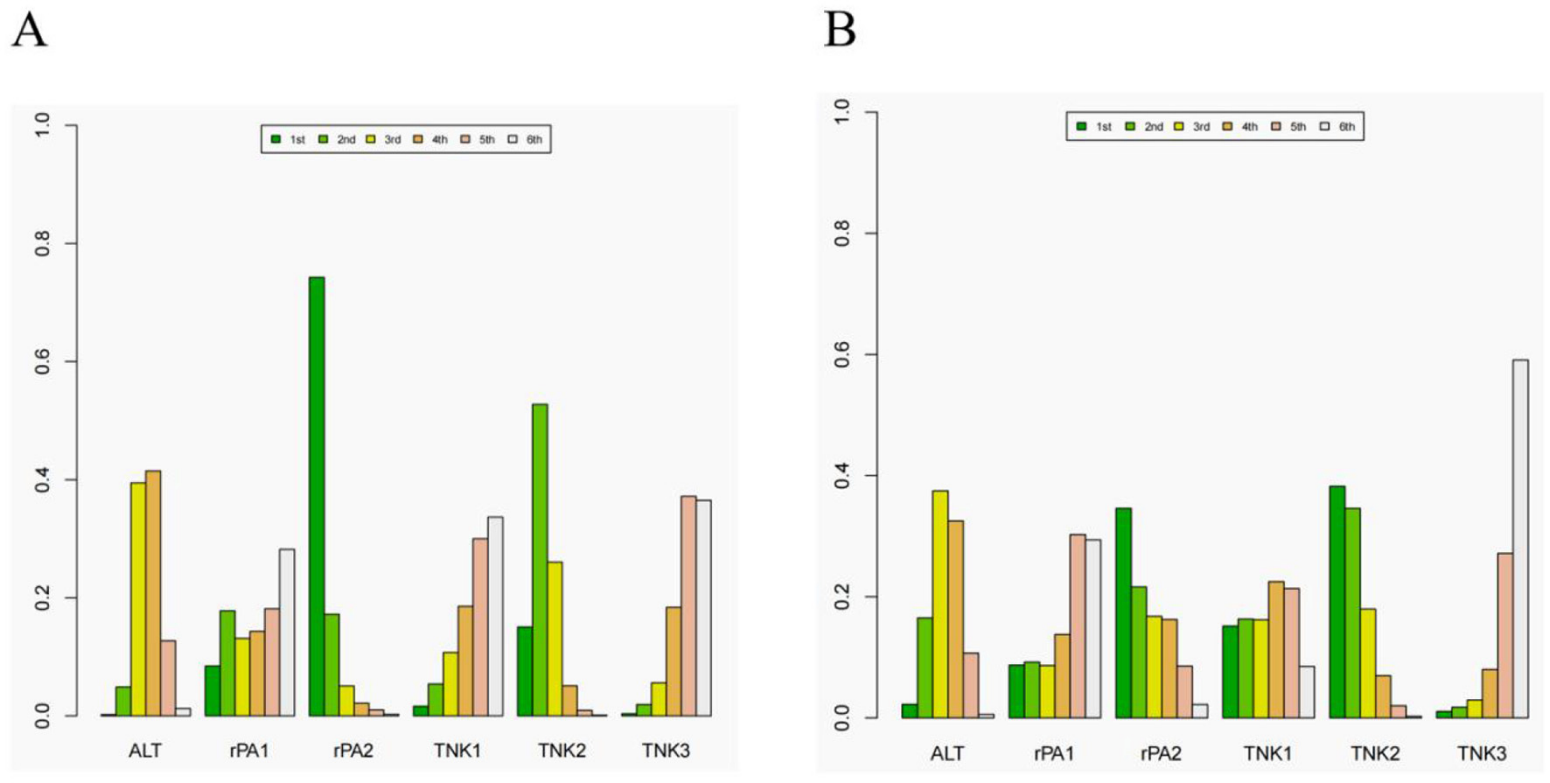
The rank of different dose of the tenecteplase and reteplase on excellent and good functional outcome at 90 days. **(A)** the rank of thrombolytic therapy on excellent functional outcome at 90 days. **(B)** the rank of thrombolytic therapy on good functional outcome at 90 days. ALT= 0.9 mg/kg alteplase, rPA1= 12 mg + 12 mg reteplase, rPA2= 18 mg + 18 mg reteplase, TNK1, 0.25 mg/kg tenecteplase; TNK2, 0.25 mg/kg tenecteplase; TNK3, 0.4 mg/kg tenecteplase.

A network meta-analysis of different doses of tenecteplase and reteplase was conducted. For excellent functional outcome at 90 days, the efficacy ranking was: 18 mg + 18 mg reteplase > 0.25 mg/kg tenecteplase > 0.9 mg/kg alteplase > 0.4 mg/kg tenecteplase > 0.1 mg/kg tenecteplase > 12 mg + 12 mg reteplase. For good functional outcome at 90 days, the ranking was: 0.25 mg/kg tenecteplase > 18 mg + 18 mg reteplase > 0.9 mg/kg alteplase > 0.1 mg/kg tenecteplase > 12 mg + 12 mg reteplase > 0.4 mg/kg tenecteplase (Figure 6). Compared to 0.9 mg/kg alteplase, patients treated with 0.25 mg/kg tenecteplase (excellent: OR 1.2, 95% CI: 0.94–1.7; good: OR 1.3, 95% CI: 0.79–2.5) and 18 mg + 18 mg reteplase (excellent: OR 1.6, 95% CI: 0.91–2.5; good: OR 1.2, 95% CI: 0.42–3.5) had better functional outcomes at 90 days (Supplementary Figures S3, S4).

### 3.4 Harms

**Figure 7:**
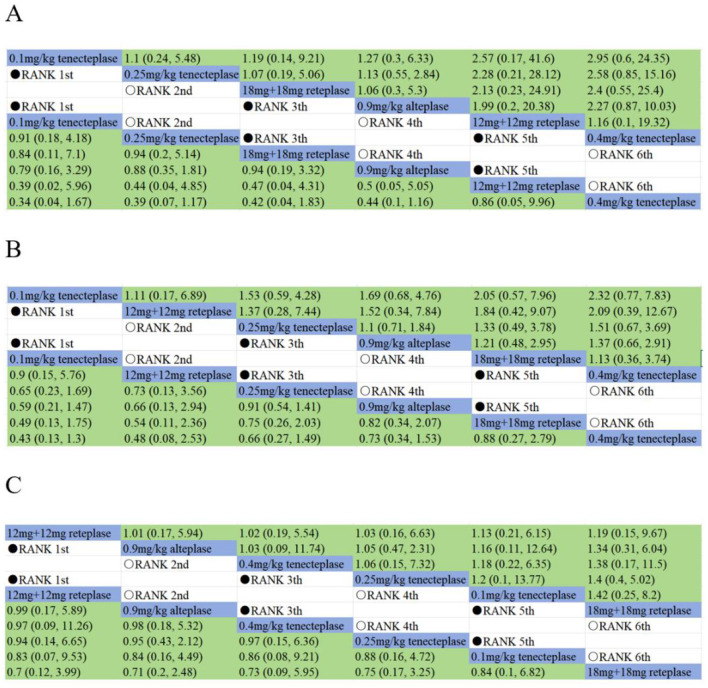
Summary of target safety outcomes by bayesian network meta-analysis, including **(A)** symptomatic intracranial haemorrhage, **(B)** death within 90 days and **(C)** serious adverse events.

All RCTs reported sICH and death within 90 days, although no sICH cases occurred in the TASTE-A trial. Eight RCTs reported SAEs. The risk of sICH ranked from lowest to highest as follows: 0.1 mg/kg tenecteplase > 0.25 mg/kg tenecteplase > 0.9 mg/kg alteplase > 18 mg + 18 mg reteplase > 12 mg + 12 mg reteplase > 0.4 mg/kg tenecteplase (Figure 7A). Both 0.1 mg/kg tenecteplase (OR 0.78, 95% CI: 0.15–3.2) and 0.25 mg/kg tenecteplase (OR 0.88, 95% CI: 0.35–1.8) had lower risks compared to alteplase, as shown in Supplementary Figure S5.

The risk of death within 90 days ranked as follows: 0.1 mg/kg tenecteplase > 12 mg + 12 mg reteplase > 0.25 mg/kg tenecteplase > 0.9 mg/kg alteplase > 18 mg + 18 mg reteplase > 0.4 mg/kg tenecteplase (Figure 7B). Here, 0.1 mg/kg tenecteplase (OR 0.59, 95% CI: 0.21–1.5), 12 mg + 12 mg reteplase (OR 0.66, 95% CI: 0.13–3.9), and 0.25 mg/kg tenecteplase (OR 0.91, 95% CI: 0.54–1.4) had lower risks compared to alteplase, as shown in Supplementary Figure S6.

The risk of SAEs ranked as follows: 12 mg + 12 mg reteplase > 0.25 mg/kg tenecteplase > 0.4 mg/kg tenecteplase > 0.9 mg/kg alteplase > 0.1 mg/kg tenecteplase > 18 mg + 18 mg reteplase (Figure 7C). The risks for 12 mg + 12 mg reteplase (OR 0.99, 95% CI: 0.17–1.9), 0.25 mg/kg tenecteplase (OR 1.0, 95% CI: 0.47–2.3), and 0.4 mg/kg tenecteplase (OR 1.0, 95% CI: 0.19–5.5) were as shown in Supplementary Figure S7.

### 3.5 Sensitivity and subgroup analyses

We performed subgroup analyses based on different patient baselines in the included RCTs. All RCTs had a higher proportion of male patients (>50%), showing no gender-based differences. Regarding mean age, only the TRACE trial (64.5 years) for tenecteplase had a mean participant age < 65 years. For reteplase, both the RAISE (63 years) and Li2024 (62.5 years) trials had mean participant ages < 65 years. Based on race, the TRACE and TRACE-2 trials, RAISE, and Li2024 (conducted in China) included Asian patients, while other RCTs predominantly included Caucasians. Stratifying by NHISS baseline, the NOR-TEST trial had a low NHISS score (< 5), the Campbell2018 trial had a moderate-high NHISS score (15–20), and other RCTs had moderate NHISS scores (5–15). Due to limitations in the number of RCTs and lack of data, we conducted subgroup analyses only for race and mean age, including tenecteplase (not dose-stratified, Supplementary Tables S5, S6), 0.25 mg/kg tenecteplase, and 18 mg + 18 mg reteplase (the most effective doses in previous analyses, Supplementary Tables S7, S8).

#### 3.5.1 Type of age

We stratified by a threshold mean age of 65 years, dividing into < 65 years and ≥65 years groups. For patients with a mean age < 65 years, tenecteplase showed no significant difference compared to alteplase (excellent: OR 0.98, 95% CI: 0.54–1.78, *P* = 0.98; good: OR 0.87, 95% CI: 0.45–1.68, *P* = 0.68). For patients with a mean age ≥65 years, tenecteplase also showed no significant difference compared to alteplase (excellent: OR 1.05, 95% CI: 0.93–1.18, P=0.44; good: OR 1.04, 95% CI: 0.81–1.34, *P* = 0.74) (Supplementary Table S5). Comparing 0.25 mg/kg tenecteplase to 0.9 mg/kg alteplase: for patients with a mean age < 65 years (excellent: OR 1.09, 95% CI: 0.52–2.3, *P* = 0.82; good: OR 1.04, 95% CI: 0.46–2.37, *P* = 0.92), and for patients with a mean age ≥65 years (excellent: OR 1.12, 95% CI: 0.98–1.29, *P* = 0.1; good: OR 1.23, 95% CI: 0.88–1.77, *P* = 0.22) (Supplementary Table S7). Regardless of average age, 0.25 mg/kg tenecteplase was the most effective tenecteplase dose, and 18 mg + 18 mg reteplase was the most effective reteplase dose (Supplementary Tables S5, S7).

#### 3.5.2 Type of ethnicity

Due to the inclusion of only Asian patients in reteplase-related RCTs, we conducted subgroup analysis for tenecteplase vs. alteplase based on ethnicity. For excellent functional outcome at 90 days, tenecteplase in Asian (OR 1.16, 95% CI: 0.95–1.42, *P* = 0.15) and in Caucasian (OR 0.99, 95% CI: 0.86–1.44, *P* = 0.93); 0.25 mg/kg tenecteplase in Asian (OR 1.18, 95% CI: 0.96–1.45, *P* = 0.12) and in Caucasian (OR 1.08, 95% CI: 0.9–1.3, *P* = 0.39) (Supplementary Tables S5, S7). These results indicate that tenecteplase is more effective in treating Asian AIS patients compared to Caucasians, and 0.25 mg/kg tenecteplase is more effective than other doses and the standard dose of alteplase across ethnicities.

#### 3.5.3 Type of NIHSS score

We stratified patients based on the average baseline NIHSS score, following the criteria of the AcT and TRACE-2 studies (21, 23), into three groups: scores below 8, between 8 and 15, and above 15. There were 3 RCTs using tenecteplase in the group with scores below 8; 6 RCTs in the group with scores between 8 and 15; and only 1 RCT in the group with scores above 15. All RCTs involving reteplase had patients with baseline NIHSS scores below 8. Regardless of the average NIHSS score, tenecteplase showed no intergroup differences in efficacy and safety outcomes compared to alteplase across all score ranges, as detailed in Supplementary Table S11.

#### 3.5.4 Other outcomes and heterogeneity analyses

This study also provided detailed subgroup analyses of the safety outcomes for tenecteplase and reteplase based on age and ethnicity (Supplementary Tables S6, S8). Additionally, we presented the publication bias of this network meta-analysis for different outcomes using funnel plots (Supplementary Figures S8–S12). Other patient stratifications (NHISS baseline, onset-to-treatment time) and outcome indicators (major neurological improvement, any intracranial hemorrhage, any parenchymal hemorrhage) were not analyzed due to insufficient data. No adjustments for other potential sources of heterogeneity were made due to a lack of power.

## 4 Discussion

This study represents the first network meta-analysis to simultaneously compare the efficacy and safety of reteplase, alteplase, and tenecteplase for treating acute ischemic stroke. By including 12 RCTs encompassing 6,633 patients (2,661 tenecteplase, 833 reteplase, and 3,139 alteplase), we assessed the benefits and risks of thrombolytic treatment with different doses of these agents. Preliminary analysis indicates that reteplase outperforms alteplase in achieving excellent functional outcomes at 90 days, while tenecteplase and alteplase show no significant differences. For other outcomes, including good functional outcomes at 90 days, symptomatic intracranial hemorrhage, death within 90 days, and serious adverse events, reteplase and tenecteplase demonstrated no significant differences compared to alteplase. Dose-specific analysis revealed that 18 mg + 18 mg reteplase and 0.25 mg/kg tenecteplase provided higher probabilities of achieving excellent/good functional outcomes at 90 days compared to 0.9 mg/kg alteplase, with 0.25 mg/kg tenecteplase showing lower risks of sICH, death within 90 days, and SAEs. Subgroup analysis by mean age and ethnicity confirmed the superior efficacy of 0.25 mg/kg tenecteplase regardless of race or age.

Despite the current lack of consensus on the optimal dose of tenecteplase for AIS, previous meta-analyses have supported 0.25 mg/kg as the most effective dose, aligning with our findings (9, 12, 27). Two RCTs demonstrated that 0.4 mg/kg tenecteplase provided no additional benefits over 0.9 mg/kg alteplase but increased the incidence of mortality and hemorrhagic events (16, 28). Our study found that 0.1 mg/kg tenecteplase was less effective than 0.9 mg/kg alteplase for 90-day functional outcomes, likely due to underdosing (29). Reteplase, traditionally used for acute myocardial infarction, has shown efficacy comparable to alteplase in trials such as GUSTO III and RAPID II (30, 31). Li's RCTs have extended the use of reteplase to AIS, suggesting that 18 mg + 18 mg reteplase is a suitable dose (25, 26). Further high-quality trials are needed to determine the optimal reteplase dose and its efficacy relative to alteplase.

Simultaneously, the thrombolytic time window for alteplase has been extensively investigated. A recent TRACE-III study indicated that the treatment time window for tenecteplase could be extended to 24 h, which resulted in an increased risk of bleeding but did not elevate the incidence of serious clinical adverse events (32). For tenecteplase and reteplase, most evidence suggests optimal thrombolysis occurs within 4.5 h; however, further research is required to ascertain whether these thrombolytic agents possess longer therapeutic windows.

Key indicators for evaluating thrombolytic efficacy include the rate of complete or partial recanalization within 24 h and major neurological improvement at 24 h (33). Previous meta-analyses have shown higher rates of successful recanalization with tenecteplase compared to alteplase (34, 35). Tenecteplase has demonstrated comparable or superior outcomes for major neurological improvement at 24 h. Some RCTs have introduced new metrics such as the Barthel Index score at 90 days to assess patient recovery, providing alternative perspectives on thrombolysis results in AIS patients (23, 24, 36). Additionally, the high cost of alteplase may limit its clinical use, whereas tenecteplase offers a cost advantage (37, 38). However, there is currently a lack of cost-effectiveness analysis comparing reteplase and alteplase for AIS treatment.

## 5 Strength and limitation

1. The two RCTs involving reteplase exclusively included Chinese stroke patients, which may introduce racial bias. Further trials focusing on Caucasian and African populations are necessary.
2. The primary patient population in RCTs conducted in Western countries is predominantly Caucasian (though not exclusively), while the majority of RCTs conducted in Asian regions involve Asian populations. This demographic difference may introduce a certain degree of bias in the description of our results. Furthermore, we believe that including an analysis by ethnicity in future RCTs could help mitigate this bias and enhance the generalizability of the study findings.
3. The small number of studies and limited sample sizes in some trials may introduce errors in our results, highlighting the need for larger-scale RCTs.
4. There are no direct comparison trials between tenecteplase and reteplase. Due to heterogeneity in the trial populations, the results from indirect comparisons have inherent limitations.
5. Different RCTs used varying scales for outcome measures, such as SITS-MOS and ECASS II for sICH, potentially introducing bias.

## 6 Conclusion

Using systematic review and meta-analysis, this study investigated the effectiveness of different thrombolytic agents in improving functional outcomes in AIS patients. We compared the efficacy of alteplase, tenecteplase, and reteplase, incorporating dose-specific and subgroup analyses. The findings indicate that tenecteplase and reteplase, in addition to alteplase, are effective treatment options for AIS. Specifically, 0.25 mg/kg tenecteplase and 18 mg + 18 mg reteplase demonstrated higher probabilities of achieving excellent/good functional outcomes at 90 days compared to 0.9 mg/kg alteplase. Furthermore, 0.25 mg/kg tenecteplase showed superior safety compared to alteplase and 18 mg + 18 mg reteplase. These results provide valuable guidance for clinicians in selecting thrombolytic agents for AIS treatment.

## Data Availability

The original contributions presented in the study are included in the article/Supplementary material, further inquiries can be directed to the corresponding author.

## References

[1] Berge E Whiteley W Audebert H De Marchis GM Fonseca AC Padiglioni C . European Stroke Organisation (ESO) guidelines on intravenous thrombolysis for acute ischaemic stroke. Eur Stroke J. (2021) 6:I–lxii. 10.1177/2396987321989865 33817340 PMC7995316

[2] Ospel JM Holodinsky JK Goyal M. Management of acute ischemic stroke due to large-vessel occlusion: JACC focus seminar. J Am Coll Cardiol. (2020) 75:1832–43. 10.1016/j.jacc.2019.10.034 32299595

[3] Powers WJ Rabinstein AA Ackerson T Adeoye OM Bambakidis NC Becker K . Guidelines for the early management of patients with acute ischemic stroke: 2019 update to the 2018 guidelines for the early management of acute ischemic stroke: a guideline for healthcare professionals from the American Heart Association/American Stroke Association. Stroke. (2019) 50:e344–418. 10.1161/STR.0000000000000211 31662037

[4] Coutts SB Berge E Campbell BC Muir KW Parsons MW. Tenecteplase for the treatment of acute ischemic stroke: A review of completed and ongoing randomized controlled trials. Int J Stroke. (2018) 13:885–92. 10.1177/1747493018790024 30035698

[5] Alamowitch S Turc G Palaiodimou L Bivard A Cameron A De Marchis GM . European Stroke Organisation (ESO) expedited recommendation on tenecteplase for acute ischaemic stroke. Eur Stroke J. (2023) 8:8–54. 10.1177/23969873221150022 37021186 PMC10069183

[6] Noble S McTavish D. Reteplase. A review of its pharmacological properties and clinical efficacy in the management of acute myocardial infarction. Drugs. (1996) 52:589–605. 10.2165/00003495-199652040-00012 8891469

[7] Smalling RW Bode C Kalbfleisch J Sen S Limbourg P Forycki F . More rapid, complete, and stable coronary thrombolysis with bolus administration of reteplase compared with alteplase infusion in acute myocardial infarction. RAPID investigators. Circulation. (1995) 91:2725–32. 10.1161/01.CIR.91.11.2725 7758177

[8] You S Saxena A Wang X Tan W Han Q Cao Y . Efficacy and safety of intravenous recombinant tissue plasminogen activator in mild ischaemic stroke: a meta-analysis. Stroke Vasc Neurol. (2018) 3:22–7. 10.1136/svn-2017-000106 29600004 PMC5870640

[9] Huang J Zheng H Zhu X Zhang K Ping X. Tenecteplase versus alteplase for the treatment of acute ischemic stroke: a meta-analysis of randomized controlled trials. Ann Med. (2024) 56:2320285. 10.1080/07853890.2024.2320285 38442293 PMC10916912

[10] Page MJ McKenzie JE Bossuyt PM Boutron I Hoffmann TC Mulrow CD . The PRISMA 2020 statement: an updated guideline for reporting systematic reviews. BMJ. (2021) 372:n71. 10.1136/bmj.n71 33782057 PMC8005924

[11] Rose D Cavalier A Kam W Cantrell S Lusk J Schrag M . Complications of intravenous tenecteplase versus alteplase for the treatment of acute ischemic stroke: a systematic review and meta-analysis. Stroke. (2023) 54:1192–204. 10.1161/STROKEAHA.122.042335 36951049 PMC10133185

[12] Ma P Zhang Y Chang L Li X Diao Y Chang H . Tenecteplase vs. alteplase for the treatment of patients with acute ischemic stroke: a systematic review and meta-analysis. J Neurol. (2022) 269:5262–71. 10.1007/s00415-022-11242-4 35776193

[13] Higgins JP Altman DG Gøtzsche PC Jüni P Moher D Oxman AD . The Cochrane Collaboration's tool for assessing risk of bias in randomised trials. BMJ. (2011) 343:d5928. 10.1136/bmj.d5928 22008217 PMC3196245

[14] White IR Barrett JK Jackson D Higgins JP. Consistency and inconsistency in network meta-analysis: model estimation using multivariate meta-regression. Res Synth Methods. (2012) 3:111–25. 10.1002/jrsm.1045 26062085 PMC4433771

[15] Haley EC Thompson JL Grotta JC Lyden PD Hemmen TG Brown DL . Phase IIB/III trial of tenecteplase in acute ischemic stroke: results of a prematurely terminated randomized clinical trial. Stroke. (2010) 41:707–11. 10.1161/STROKEAHA.109.572040 20185783 PMC2860601

[16] Bivard A Zhao H Churilov L Campbell BCV Coote S Yassi N . Comparison of tenecteplase with alteplase for the early treatment of ischaemic stroke in the Melbourne Mobile Stroke Unit (TASTE-A): a phase 2, randomised, open-label trial. Lancet Neurol. (2022) 21:520–7. 10.1016/S1474-4422(22)00171-5 35525251

[17] Campbell BCV Mitchell PJ Churilov L Yassi N Kleinig TJ Dowling RJ . Tenecteplase versus alteplase before thrombectomy for ischemic stroke. N Engl J Med. (2018) 378:1573–82. 29694815 10.1056/NEJMoa1716405

[18] Huang X Cheripelli BK Lloyd SM Kalladka D Moreton FC Siddiqui A . Alteplase versus tenecteplase for thrombolysis after ischaemic stroke (ATTEST): a phase 2, randomised, open-label, blinded endpoint study. Lancet Neurol. (2015) 14:368–76. 10.1016/S1474-4422(15)70017-7 25726502

[19] Li S Pan Y Wang Z Liang Z Chen H Wang D . Safety and efficacy of tenecteplase versus alteplase in patients with acute ischaemic stroke (TRACE): a multicentre, randomised, open label, blinded-endpoint (PROBE) controlled phase II study. Stroke Vasc Neurol. (2022) 7:47–53. 10.1136/svn-2021-000978 34429364 PMC8899644

[20] Logallo N Novotny V Assmus J Kvistad CE Alteheld L Rønning OM . Tenecteplase versus alteplase for management of acute ischaemic stroke (NOR-TEST): a phase 3, randomised, open-label, blinded endpoint trial. Lancet Neurol. (2017) 16:781–8. 10.1016/S1474-4422(17)30253-3 28780236

[21] Menon BK Buck BH Singh N Deschaintre Y Almekhlafi MA Coutts SB . Intravenous tenecteplase compared with alteplase for acute ischaemic stroke in Canada (AcT): a pragmatic, multicentre, open-label, registry-linked, randomised, controlled, non-inferiority trial. Lancet. (2022) 400:161–9. 10.1016/S0140-6736(22)01054-6 35779553

[22] Parsons M Spratt N Bivard A Campbell B Chung K Miteff F . A randomized trial of tenecteplase versus alteplase for acute ischemic stroke. N Engl J Med. (2012) 366:1099–107. 10.1056/NEJMoa1109842 22435369

[23] Wang Y Li S Pan Y Li H Parsons MW Campbell BCV . Tenecteplase versus alteplase in acute ischaemic cerebrovascular events (TRACE-2): a phase 3, multicentre, open-label, randomised controlled, non-inferiority trial. Lancet. (2023) 401:645–54. 36774935 10.1016/S0140-6736(22)02600-9

[24] Kvistad CE Næss H Helleberg BH Idicula T Hagberg G Nordby LM . Tenecteplase versus alteplase for the management of acute ischaemic stroke in Norway (NOR-TEST 2, part A): a phase 3, randomised, open-label, blinded endpoint, non-inferiority trial. Lancet Neurol. (2022) 21:511–9. 10.1016/S1474-4422(22)00124-7 35525250

[25] Li S Gu HQ Li H Wang X Jin A Guo S . Reteplase versus alteplase for acute ischemic stroke. N Engl J Med. (2024) 390:2264–73. 10.1056/NEJMoa2400314 38884332

[26] Li S Wang X Jin A Liu G Gu H Li H . Safety and efficacy of reteplase versus alteplase for acute ischemic stroke: a phase 2 randomized controlled trial. Stroke. (2024) 55:366–75. 10.1161/STROKEAHA.123.045193 38152962

[27] Nair R Wagner AN Buck BH. Advances in the management of acute ischemic stroke. Curr Opin Neurol. (2023) 36:147–54. 10.1097/WCO.0000000000001136 36762632

[28] Campbell BCV Mitchell PJ Churilov L Yassi N Kleinig TJ Dowling RJ . Effect of intravenous tenecteplase dose on cerebral reperfusion before thrombectomy in patients with large vessel occlusion ischemic stroke: the extend-ia tnk part 2 randomized clinical trial. JAMA. (2020) 323:1257–65. 10.1001/jama.2020.1511 32078683 PMC7139271

[29] Warach SJ Dula AN Milling TJ. Tenecteplase thrombolysis for acute ischemic stroke. Stroke. (2020) 51:3440–51. 10.1161/STROKEAHA.120.029749 33045929 PMC7606819

[30] Global Use of Strategies to Open Occluded Coronary Arteries (GUSTO III) Investigators. A comparison of reteplase with alteplase for acute myocardial infarction. N Engl J Med. (1997) 337:1118–23. 10.1056/NEJM199710163371603 9340503

[31] Bode C Smalling RW Berg G Burnett C Lorch G Kalbfleisch JM . Randomized comparison of coronary thrombolysis achieved with double-bolus reteplase (recombinant plasminogen activator) and front-loaded, accelerated alteplase (recombinant tissue plasminogen activator) in patients with acute myocardial infarction. The RAPID II investigators. Circulation. (1996) 94:891–8. 10.1161/01.CIR.94.5.891 8790022

[32] Xiong Y Campbell BCV Schwamm LH Meng X Jin A Parsons MW . Tenecteplase for ischemic stroke at 45 to 24 hours without thrombectomy. N Engl J Med. (2024) 391:203–12. 10.1056/NEJMoa2402980 38884324

[33] Kobeissi H Ghozy S Turfe B Bilgin C Kadirvel R Kallmes DF . Tenecteplase vs. alteplase for treatment of acute ischemic stroke: A systematic review and meta-analysis of randomized trials. Front Neurol. (2023) 14:1102463. 10.3389/fneur.2023.1102463 36756249 PMC9900099

[34] Thelengana A Radhakrishnan DM Prasad M Kumar A Prasad K. Tenecteplase versus alteplase in acute ischemic stroke: systematic review and meta-analysis. Acta Neurol Belg. (2019) 119:359–67. 10.1007/s13760-018-0933-9 29728903

[35] Walton MN Hamilton LA Salyer S Wiseman BF Forster AM Rowe AS. Major bleeding postadministration of tenecteplase versus alteplase in acute ischemic stroke. Ann Pharmacother. (2023) 57:535–43. 10.1177/10600280221120211 36004394

[36] Gurková E Štureková L Mandysová P Šanák D. Factors affecting the quality of life after ischemic stroke in young adults: a scoping review. Health Qual Life Outc. (2023) 21:4. 10.1186/s12955-023-02090-5 36653785 PMC9850784

[37] Gao L Parsons M Churilov L Zhao H Campbell BC Yan B . Cost-effectiveness of tenecteplase versus alteplase for stroke thrombolysis evaluation trial in the ambulance. Eur Stroke J. (2023) 8:448–55. 10.1177/23969873231165086 37231684 PMC10334173

[38] Seyedroudbari A Kessler ER Mooss AN Wundeman RL Bala M Hilleman DE. Time to treatment and cost of thrombolysis: a multicenter comparison of tPA and rPA. J Thromb Thrombolysis. (2000) 9:303–8. 10.1023/A:1018797411812 10728031

