# Supplementary material for: Thrombolytic therapy for patients with acute ischemic stroke: systematic review and network meta-analysis of randomized trials
Source: Front Neurol. 2025 Jan 7;15:1490476. doi: 10.3389/fneur.2024.1490476 (PMC11746078; doi:10.3389/fneur.2024.1490476)
Supplement: Supplementary file 1 [file Supplementary_file_1.docx]

Thrombolytic therapy for patients with acute ischemic stroke: systematic review and network meta-analysis of randomized trials

| **Table of Contents** | | |
| --- | --- | --- |
| Title | Content | page |
| Table S1 | The comparisons between this review and the original PROSPERO protocol | 2 |
| Table S2 | PRISMA Checklist of Items to Include When Reporting a Systematic Review Involving a Meta-analysis | 2-5 |
| Table S3 | Criteria for Inclusion and Exclusion | 6 |
| Table S4 | Literature Search Strategy | 7 |
| Table S5 | The definitions of efficacy and safety outcomes in Randomized Controlled Trials | 8-9 |
| Table S6 | The table detailing thrombus locations / stroke subtypes, along with information on mechanical thrombectomy. | 10 |
| Table S7 | The subgroup analyses of tenecteplase and reteplase on effecacy outcomes | 11 |
| Table S8 | The subgroup analyses of tenecteplase and reteplase on safety outcomes | 11-12 |
| Table S9 | The subgroup analyses of 0.25mg/kg tenecteplase and 18mg+18mg reteplase on effecacy outcomes | 12 |
| Table S10 | The subgroup analyses of 0.25mg/kg tenecteplase and 18mg+18mg reteplase on safety outcomes | 12-13 |
| Table S11 | The influence of the average baseline NIHSS score on the efficacy and safety of thrombolytic drugs | 14 |
| Figure S1 | Network plot of studies for different outcomes. | 15 |
| Figure S2 | The contribution to the network estimates. | 15 |
| Figure S3 | The forest of different dose of the tenecteplase and reteplase on excellent functional outcome at 90 days. | 16 |
| Figure S4 | The forest of different dose of the tenecteplase and reteplase on good functional outcome at 90 days. | 17 |
| Figure S5 | The forest of different dose of the tenecteplase and reteplase on symptomatic intracranial haemorrhage. | 17 |
| Figure S6 | The forest of different dose of the tenecteplase and reteplase on death within 90 days. | 17 |
| Figure S7 | The forest of different dose of the tenecteplase and reteplase on serious adverse events. | 18 |
| Figure S8 | Funnel plot for different dose of the tenecteplase and reteplase on excellent functional outcome at 90 days. | 18 |
| Figure S9 | Funnel plot for different dose of the tenecteplase and reteplase on good functional outcome at 90 days. | 19 |
| Figure S10 | Funnel plot for different dose of the tenecteplase and reteplase on symptomatic intracranial haemorrhage. | 19 |
| Figure S11 | Funnel plot for different dose of the tenecteplase and reteplase on death within 90 days. | 20 |
| Figure S12 | Funnel plot for different dose of the tenecteplase and reteplase on serious adverse events. | 20 |

Table S1 The comparisons between this review and the original PROSPERO protocol

| The section | Network meta-analysis | PROSPERO protocol |
| --- | --- | --- |
| Population | Acute ischemic stroke (AIS) patients (***≥18 years), who were eligible***  ***for intravenous thrombolysis using standard guidelinebased criteria***. | AIS patients |
| Intervention | Different ***doses*** of thrombolytic therapy (tenecteplase ***(0.1 mg/kg, 0.25 mg/kg, 0.4 mg/kg),*** reteplase(***12mg+12mg, 18mg+18mg***)) | Different thrombolytic therapy (tenecteplase, reteplase) |
| Comparison | The standard dose of alteplase(0.9mg/kg) | The standard dose of alteplase |
| Outcomes | Excellent and good functional outcome at 90 days；death at 90 days; ***symptomatic intracranial haemorrhage(sICH), serious adverse events (SAEs).*** | Excellent and good functional outcome at 90 days；death at 90 days. |
| Study design | Phase II or Phase III randomized controlled trials | Phase II or Phase III RCTs |

Table S2 PRISMA Checklist of Items to Include When Reporting a Systematic Review Involving a Meta-analysis

| **Section/Topic** | **Item #** | **Checklist Item** | **Reported on Page #** |
| --- | --- | --- | --- |
| **TITLE** |  |  |  |
| Title | 1 | Identify the report as a systematic review *incorporating*  *anetwork meta-analysis (or related form of meta-analysis).* | **1** |
|  |  |  |  |
| **ABSTRACT** |  |  |  |
| Structured summary | 2 | Provide a structured summary including, as applicable:  **Background:** main objectives  **Methods:** data sources; study eligibility criteria, participants, and interventions; study appraisal; and *synthesis methods, such as network meta-analysis.*  **Results:** number of studies and participants identified; summary estimates with corresponding confidence/credible intervals; *treatment rankings may also be discussed. Authors may choose to summarize pairwise comparisons against a chosen treatment included in their analyses for brevity.*  **Discussion/Conclusions:** limitations; conclusions and implications of findings.  **Other:** systematic review registration number with registry name. | 1-2 |
|  |  |  |  |
| **INTRODUCTION** |  |  |  |
| Rationale | 3 | Describe the rationale for the review in the context of what is already known*, including mention of why a network meta-analysis has been conducted.* | **2** |
| Objectives | 4 | Provide an explicit statement of questions being addressed, with reference to participants, interventions, comparisons, outcomes, and study design (PICOS). | 2 |
|  |  |  |  |
| **METHODS** |  |  |  |
| Protocol and registration | 5 | Indicate whether a review protocol exists and if and where it can be accessed (e.g., Web address); and, if available, provide registration information, including registration number. | 3 |
| Eligibility criteria | 6 | Specify study characteristics (e.g., PICOS, length of follow-up) and report characteristics (e.g., years considered, language, publication status) used as criteria for eligibility, giving rationale. *Clearly describe eligible treatments included in the treatment network, and note whether any have been clustered or merged into the same node (with justification).* | **3** |
| Information sources | 7 | Describe all information sources (e.g., databases with dates of coverage, contact with study authors to identify additional studies) in the search and date last searched. | **3** |
| Search | 8 | Present full electronic search strategy for at least one database, including any limits used, such that it could be repeated. | 3，Supplementary  TableS3 |
| Study selection | 9 | State the process for selecting studies (i.e., screening, eligibility, included in systematic review, and, if applicable, included in the meta-analysis). | **3,** Supplementary  TableS2 |
| Data collection process | 10 | Describe method of data extraction from reports (e.g., piloted forms, independently, in duplicate) and any processes for obtaining and confirming data from investigators. | 3 |
| Data items | 11 | List and define all variables for which data were sought (e.g., PICOS, funding sources) and any assumptions and simplifications made. | **3** |
| **Geometry of the network** | **S1** | Describe methods used to explore the geometry of the treatment network under study and potential biases related to it. This should include how the evidence base has been graphically summarized for presentation, and what characteristics were compiled and used to describe the evidence base to readers. | 4 |
| Risk of bias within individual studies | 12 | Describe methods used for assessing risk of bias of individual studies (including specification of whether this was done at the study or outcome level), and how this information is to be used in any data synthesis. | 4 |
| Summary measures | 13 | State the principal summary measures (e.g., risk ratio, difference in means). *Also describe the use of additional summary measures assessed, such as treatment rankings and surface under the cumulative ranking curve (SUCRA) values, as well as modified approaches used to present summary findings from meta-analyses.* | 3-4 |
| Planned methods of analysis | 14 | Describe the methods of handling data and combining results of studies for each network meta-analysis. This should include, but not be limited to:   - *Handling of multi-arm trials;* - *Selection of variance structure;* - *Selection of prior distributions in Bayesian analyses; and* - *Assessment of model fit.* | **3** |
| **Assessment of Inconsistency** | **S2** | Describe the statistical methods used to evaluate the agreement of direct and indirect evidence in the treatment network(s) studied. Describe efforts taken to address its presence when found. | 3-4 |
| Risk of bias across studies | 15 | Specify any assessment of risk of bias that may affect the cumulative evidence (e.g., publication bias, selective reporting within studies). | **4** |
| Additional analyses | 16 | Describe methods of additional analyses if done, indicating which were pre-specified. This may include, but not be limited to, the following:   - Sensitivity or subgroup analyses; - Meta-regression analyses; - *Alternative formulations of the treatment network; and* - *Use of alternative prior distributions for Bayesian analyses (if applicable).* | 3-4 |
|  |  |  |  |
| **RESULTS†** |  |  |  |
| Study selection | 17 | Give numbers of studies screened, assessed for eligibility, and included in the review, with reasons for exclusions at each stage, ideally with a flow diagram. | **5** |
| **Presentation of network structure** | **S3** | Provide a network graph of the included studies to enable visualization of the geometry of the treatment network. | 5 |
| **Summary of network geometry** | **S4** | Provide a brief overview of characteristics of the treatment network. This may include commentary on the abundance of trials and randomized patients for the different interventions and pairwise comparisons in the network, gaps of evidence in the treatment network, and potential biases reflected by the network structure. | **5-6** |
| Study characteristics | 18 | For each study, present characteristics for which data were extracted (e.g., study size, PICOS, follow-up period) and provide the citations. | Table 1, Table 2, |
| Risk of bias within studies | 19 | Present data on risk of bias of each study and, if available, any outcome level assessment. | **6, Figure 3** |
| Results of individual studies | 20 | For all outcomes considered (benefits or harms), present, for each study: 1) simple summary data for each intervention group, and 2) effect estimates and confidence intervals. *Modified approaches may be needed to deal with information from larger networks.* | 6, Table S4 |
| Synthesis of results | 21 | Present results of each meta-analysis done, including confidence/credible intervals. *In larger networks, authors may focus on comparisons versus a particular comparator (e.g. placebo or standard care), with full findings presented in an appendix. League tables and forest plots may be considered to summarize pairwise comparisons.* If additional summary measures were explored (such as treatment rankings), these should also be presented. | **6-10** |
| **Exploration for inconsistency** | **S5** | Describe results from investigations of inconsistency. This may include such information as measures of model fit to compare consistency and inconsistency models, *P* values from statistical tests, or summary of inconsistency estimates from different parts of the treatment network. | 10 |
| Risk of bias across studies | 22 | Present results of any assessment of risk of bias across studies for the evidence base being studied. | **7, Figure 3** |
| Results of additional analyses | 23 | Give results of additional analyses, if done (e.g., sensitivity or subgroup analyses, meta-regression analyses*, alternative network geometries studied, alternative choice of prior distributions for Bayesian analyses,* and so forth). | 10 |
|  |  |  |  |
| **DISCUSSION** |  |  |  |
| Summary of evidence | 24 | Summarize the main findings, including the strength of evidence for each main outcome; consider their relevance to key groups (e.g., healthcare providers, users, and policy-makers). | **10-11** |
| Limitations | 25 | Discuss limitations at study and outcome level (e.g., risk of bias), and at review level (e.g., incomplete retrieval of identified research, reporting bias). *Comment on the validity of the assumptions, such as transitivity and consistency. Comment on any concerns regarding network geometry (e.g., avoidance of certain comparisons).* | 11 |
| Conclusions | 26 | Provide a general interpretation of the results in the context of other evidence, and implications for future research. | **11** |
|  |  |  |  |
| **FUNDING** |  |  |  |
| Funding | 27 | Describe sources of funding for the systematic review and other support (e.g., supply of data); role of funders for the systematic review. This should also include information regarding whether funding has been received from manufacturers of treatments in the network and/or whether some of the authors are content experts with professional conflicts of interest that could affect use of treatments in the network. | ***12*** |

PICOS = population, intervention, comparators, outcomes, study design.

* Text in italics indicate S wording specific to reporting of network meta-analyses that has been added to guidance from the PRISMA statement.

† Authors may wish to plan for use of appendices to present all relevant information in full detail for items in this section.

Table S3. Criteria for Inclusion and Exclusion

| **Inclusion Criteria** | 1. randomized controlled trials (RCTs) formally published in peer-reviewed journals; 2. patients in the intervention group were treated with tenecteplase or reteplase, 3. patients in the comparison group were treated with alteplase;      1. at least reported one primary outcomes. |
| --- | --- |
| **Exclusion Criteria** | 1. non-English-language studies; 2. fundamental experiment studies, conference abstracts, case reports, and reviews; 3. studies without comparison group; 4. overlapping participant data |

Table S4. Literature Search Strategy

| **Table S2.Literature Search Strategy** | |
| --- | --- |
| **Pubmed** | (("tenecteplase"[Title/Abstract] OR "reteplase"[Title/Abstract] OR "alteplase"[Title/Abstract] OR "IVT"[Title/Abstract] OR "intravenous thrombolysis "[Title/Abstract]) AND ("stroke"[Title/Abstract] OR "AIS"[Title/Abstract]) AND ("random"[Title/Abstract] OR "randomized"[Title/Abstract] OR "randomised"[Title/Abstract])) |
| **Web of Science** | #1:TS=(("tenecteplase" OR "reteplase" OR "alteplase" OR "IVT" OR "intravenous thrombolysis "  )  #2:TS=("stroke" OR "AIS" OR "acute ischemic stroke")  #3:TS=(( randomized controlled trial; controlled clinical trial) OR (randomized) OR (randomised) OR (placebo) OR (sham) OR (randomly) OR (trial) OR (groups))  #4: #1 AND #2  #5: #3 AND #4 |
| **Cochrane** | 1#: "tenecteplase" OR "reteplase" OR "alteplase"  2#: "rPA" OR "rt-PA" OR "ALT" OR "TNK"  3#: 1# OR 2#  4#: "stroke" OR "AIS" OR "acute ischemic stroke"  5#: "IVT" OR "intravenous thrombolysis "  6#: 4# AND 5#  7#: 3# OR 6#  8#: "random" OR "randomized" OR "randomised" OR "RCT"  9#: 7# AND 8# |
| **SCOPUS** | 1#: TITLE-ABS-KEY: (“tenecteplase" OR "reteplase" OR "alteplase" )  2#: TITLE-ABS-KEY: ("stroke" OR "AIS" OR "acute ischemic stroke")  3#: TITLE-ABS-KEY: ((randomized controlled trial; controlled clinical trial) OR (randomized) OR (randomised) OR (placebo) OR (sham) OR (randomly) OR (trial) OR (groups))  4#: TITLE-ABS-KEY: ("rPA" OR "rt-PA" OR "ALT" OR "TNK")  5#: 1# OR 4#  6#: 2# AND 3# AND 5# |
|  |  |

**Table S5 The definitions of efficacy and safety outcomes in Randomized Controlled Trials**

| **Study** | **excellent functional outcome at 90 days** | **good functional outcome at 90 days** | **Symptomatic intracranial haemorrhage** | **Death within 90 days** | **Serious adverse events** |
| --- | --- | --- | --- | --- | --- |
| Haley2010 | mRS ranking=0/1 | NA | any clinically important neurological worsening | Death from any cause within 90 days | NA |
| Parons2012 | mRS ranking=0-1 | mRS ranking=0-2 | a large parenchymal hematoma and clinical worsening (an increase in the NIHSS score of 4 or more points) | NA | NA |
| ATTEST | mRS ranking=0-1 | mRS ranking=0-2 | according to SITS-MOST criteria | Death from any cause within 90 days | AEs Grade≥3 |
| NOR-TEST | mRS ranking=0-1 | mRS ranking=0-2 | according to ECASS III criteria | death within 3 months after inclusion | mainly any types of intracranial haemorrhage |
| Campbell 2018 | mRS score of 0 to 1 or no change from baseline | mRS score of 0 to 2 or no change from baseline | according to SITS-MOST criteria | death due to any cause | Life threatening / prolonged hospitalization, results in death, medically Important |
| TRACE | mRS ≤1 point at 90 day | mRS ≤2 point at 90 day | according to ECASS III criteria | deaths from any cause | AEs Grade≥3 |
| TASTE-A | mRS 0–1 or no change from baseline at 90 days | mRS 0–2 or no change from  baseline at 90 days | according to SITS-MOST criteria | death due to any cause at 90 days | NA |
| NOR-TEST2（PARTA） | mRS score of 0–1 or a return to baseline | mRS score of 0–2 or a return to baseline | according to ECASS III criteria | death due to any cause at 90 days | NA |
| AcT | mRS score of 0–1 | mRS score of 0–2 | according to ECASS III criteria | 90-day all-cause mortality | AEs Grade≥3 (including:any types of intracranial haemorrhage) |
| TRACE-2 | mRS score of 0–1 at 90 days | mRS score of 0–2 at 90 days | according to ECASS III criteria | death from all causes within 90 days of disease | AEs Grade≥3 at 90 days |
| Li2024 | mRS score of 0–1 at 90 days | mRS score of 0–2 at 90 days | according to SITS-MOST criteria | all causes of death within 90 days | AEs Grade≥3 within 90 days |
| RAISE | mRS score of 0 or 1 at 90 days | mRS score of 0 -2 at 90 days | according to ECASS III criteria | all causes of death within 90 days | AEs Grade≥3 within 90 days |
| **mRS**:modified Rankin Scale;  **SITS-MOSTcriteria**: parenchymal haemorrhage type 2 or remote parenchymal haemorrhage type 2 on 24–48 h non-contrast CT, plus neurological deterioration of ≥4 points NIHSS score; **ECASS III criteria**: any intracerebral haemorrhage on follow-up non-contrast CT with clinical deterioration. | | | | | |

**Table S6 The statistical table detailing thrombus locations and stroke subtypes, along with information on mechanical thrombectomy.**

| **Study** | **Occlusion location** | **Mechanical thrombectomy** |
| --- | --- | --- |
| Haley2010 | according to TOAST criteria(Large vessel Atherothromboembolic, Cardioembolic, Small vessel,Unknown cause) | NA |
| Parons2012 | ACA(1%),proximal section of first segment of middle cerebral artery(32%),M1(11%),distal section of first segment of middle cerebral artery(33%),M2(11%),PCA(4%),terminal internal carotid artery(1%),none(4%) | Bridging thrombolysis plus endovascular thrombectomy was allowed but was excluded from per protocol analysis |
| ATTEST | ICA(29%),M1(46%),M2(17%),M3(3%),ACA or PCA(6%) | NA |
| NOR-TEST | according to TOAST criteria(Large vessel Atherothromboembolic(20%),Cardioembolic(tenecteplase:21%,alteplase:21%),Small vessel(tenecteplase:15%,alteplase:12%),other cause(tenecteplase:5%,alteplase:6%), Unknown cause(tenecteplase:39%,alteplase:36%)) | NA |
| Campbell 2018 | involved vessel (ICA, basilar artery, M1, M2) | 50 patients(27[27%] who received tenecteplase and 23 [23%] who received alteplase) |
| TRACE | NA | Bridging thrombolysis plus endovascular thrombectomy was allowed but was excluded from per protocol analysis. |
| TASTE-A | large vessel occlusion(alteplase:19(39%),tenecteplase:27(49%)) | 24 patients(13 [24%] who received tenecteplase and 11 [22%] who received alteplase) |
| NOR-TEST2（PARTA） | Major arterial vessel occlusion(alteplase:59(57%),tenecteplase:57(57%)) | NA |
| AcT | ICA(8.6%),M1(15.2%),M2(20.5%),Other distal occlusions(17.2%),Vertebrobasilar arterial system(4.2%),Cervical internal carotid artery(1.8%),No visible occlusions(33%) | 58 patients(31 in the reteplase group and 27 in the alteplase group) |
| TRACE-2 | NA | 51 patients(27 in the reteplase group and 24 in the alteplase group) |
| Li2024 | NA | NA |
| RAISE | NA | 45 patients (20 in the reteplase group and 25 in the alteplase group) |
| ICA=internal carotid artery.M1=middle cerebral artery M1 segment. M2=middle cerebral artery M2 segment.M3=middle cerebral artery M3 segment. ACA=anterior cerebral artery.PCA=posterior cerebral artery. | | |

**Table S7 The subgroup analyses of tenecteplase and reteplase on effecacy outcomes**

| **Outcomes** | **Comparisons*** | **Subgroups** | | **Number of  comparisons** | **P value** | **Odds ratio（95%Cl）** | **I^2^** |
| --- | --- | --- | --- | --- | --- | --- | --- |
| Excellent functional outcome at 90 days | Tenecteplase versus alteplase | Age | ≥65 years | 9 | 0.44 | 1.05(0.93,1.18) | 38% |
|  |  |  | ＜65 years | 1 | 0.98 | 0.98(0.54,1.78) | NA |
|  |  | Race | Caucasian | 8 | 0.93 | 0.99(0.86,1.14) | 37% |
|  |  |  | Asian | 2 | 0.15 | 1.16(0.95,1.42) | 0% |
|  | Reteplase versus alteplase | Age | ≥65 years | NA | NA | NA | NA |
|  |  |  | ＜65 years | 2 | 0.0002 | 1.55(1.23,1.95) | 34% |
|  |  | Race | Caucasian | NA | NA | NA | NA |
|  |  |  | Asian | 2 | 0.0002 | 1.55(1.23,1.95) | 34% |
| Good functional outcome at 90 days | Tenecteplase versus alteplase | Age | ≥65 years | 8 | 0.74 | 1.04(0.81,1.34) | 64% |
|  |  |  | ＜65 years | 1 | 0.68 | 0.87(0.45,1.68) | NA |
|  |  | Race | Caucasian | 7 | 0.71 | 1.06(0.76,1.48) | 69% |
|  |  |  | Asian | 2 | 0.78 | 1.03(0.83,1.29) | 0% |
|  | Reteplase versus alteplase | Age | ≥65 years | NA | NA | NA | NA |
|  |  |  | ＜65 years | 2 | 0.66 | 1.15(0.61,2.19) | 61% |
|  |  | Race | Caucasian | NA | NA | NA | NA |
|  |  |  | Asian | 2 | 0.66 | 1.15(0.61,2.19) | 61% |

**Table S8 The subgroup analyses of tenecteplase and reteplase on safety outcomes**

| **Outcomes** | **Comparisons*** | **Subgroups** | | **Number of  comparisons** | **P value** | **Odds ratio（95%Cl）** | **I^2^** |
| --- | --- | --- | --- | --- | --- | --- | --- |
| Symptomatic intracranial haemorrhage | Tenecteplase versus alteplase | Mean age | ≥65 years | 8 | 0.58 | 1.1(0.78,1.55) | 0% |
|  |  |  | ＜65 years | 1 | 0.64 | 1.69(0.19,14.73) | NA |
|  |  | Race | Caucasian | 7 | 0.65 | 1.1(0.74,1.62) | 0% |
|  |  |  | Asian | 2 | 0.64 | 1.17(0.6,2.28) | 0% |
|  | Reteplase versus alteplase | Mean age | ≥65 years | NA | NA | NA | NA |
|  |  |  | ＜65 years | 2 | 0.82 | 1.08(0.56,2.07) | 0% |
|  |  | Race | Caucasian | NA | NA | NA | NA |
|  |  |  | Asian | 2 | 0.82 | 1.08(0.56,2.07) | 0% |
| Death within 90 days | Tenecteplase versus alteplase | Mean age | ≥65 years | 9 | 0.68 | 0.96(0.79,1.17) | 43% |
|  |  |  | ＜65 years | 1 | 0.4 | 0.64(0.23,1.80) | NA |
|  |  | Race | Caucasian | 8 | 0.3 | 0.89(0.72,1.11) | 39% |
|  |  |  | Asian | 2 | 0.43 | 1.18(0.78,1.79) | 37% |
|  | Reteplase versus alteplase | Mean age | ≥65 years | NA | NA | NA | NA |
|  |  |  | ＜65 years | 2 | 0.53 | 1.17(0.71,1.91) | 0 |
|  |  | Race | Caucasian | NA | NA | NA | NA |
|  |  |  | Asian | 2 | 0.53 | 1.17(0.71,1.91) | 0% |
| Serious adverse event | Tenecteplase versus alteplase | Mean age | ≥65 years | 5 | 0.53 | 1.12(0.79,1.57) | 72% |
|  |  |  | ＜65 years | 1 | 0.25 | 0.66(0.32,1.34) | NA |
|  |  | Race | Caucasian | 4 | 0.56 | 1.16(0.7,1.91) | 79% |
|  |  |  | Asian | 2 | 0.92 | 1.01(0.78,1.32) | 39% |
|  | Reteplase versus alteplase | Mean age | ≥65 years | NA | NA | NA | NA |
|  |  |  | ＜65 years | 2 | 0.07 | 1.31(0.98,1.75) | 0% |
|  |  | Race | Caucasian | NA | NA | NA | NA |
|  |  |  | Asian | 2 | 0.07 | 1.31(0.98,1.75) | 0% |

**Table S9 The subgroup analyses of 0.25mg/kg tenecteplase and 18mg+18mg reteplase on effecacy outcomes**

| **Outcomes** | **Comparisons*** | **Subgroups** | | **Number of  comparisons** | **P value** | **Odds ratio（95%Cl）** | **I^2^** |
| --- | --- | --- | --- | --- | --- | --- | --- |
| Excellent functional outcome at 90 days | 0.25mg/kg Tenecteplase versus 0.9mg/kg alteplase | Mean age | ≥65 years | 7 | 0.1 | 1.12(0.98,1.29) | 19% |
|  |  |  | ＜65 years | 1 | 0.82 | 1.09(0.52,2.3) | NA |
|  |  | Race | Caucasian | 6 | 0.39 | 1.08（0.9，1.3） | 29% |
|  |  |  | Asian | 2 | 0.12 | 1.18(0.96,1.45) | 0% |
|  | 18mg+18mg Reteplase versus 0.9mg/kg alteplase | Mean age | ≥65 years | NA | NA | NA | NA |
|  |  |  | ＜65 years | 2 | ＜0.0001 | 1.6（1.27，2.02） | 0% |
|  |  | Race | Caucasian | NA | NA | NA | NA |
|  |  |  | Asian | 2 | ＜0.0001 | 1.6（1.27，2.02） | 0% |
| Good functional outcome at 90 days | 0.25mg/kg Tenecteplase versus 0.9mg/kg alteplase | Mean age | ≥65 years | 6 | 0.22 | 1.23(0.88,1.71) | 67% |
|  |  |  | ＜65 years | 1 | 0.92 | 1.04(0.46,2.37) | NA |
|  |  | Race | Caucasian | 5 | 0.19 | 1.43(0.83,2.47) | 73% |
|  |  |  | Asian | 2 | 0.65 | 1.05(0.84,1.32) | 0% |
|  | 18mg+18mg Reteplase versus 0.9mg/kg alteplase | Mean age | ≥65 years | NA | NA | NA | NA |
|  |  |  | ＜65 years | 2 | 0.01 | 1.41(1.08,1.83) | 0% |
|  |  | Race | Caucasian | NA | NA | NA | NA |
|  |  |  | Asian | 2 | 0.01 | 1.41(1.08,1.83) | 0% |

**Table S10 The subgroup analyses of 0.25mg/kg tenecteplase and 18mg+18mg reteplase on safety outcomes**

| **Outcomes** | **Comparisons*** | **Subgroups** | | **Number of  comparisons** | **P value** | **Odds ratio（95%Cl）** | **I^2^** |
| --- | --- | --- | --- | --- | --- | --- | --- |
| Symptomatic intracranial haemorrhage | 0.25mg/kg Tenecteplase versus 0.9mg/kg alteplase | Mean age | ≥65 years | 6 | 0.98 | 0.99(0.67,1.49) | 0% |
|  |  |  | ＜65 years | 1 | 0.51 | 0.34(0.01,8.5) | NA |
|  |  | Race | Caucasian | 5 | 0.98 | 0.94(0.58,1.53) | 0% |
|  |  |  | Asian | 2 | 0.89 | 1.05(0.53,2.08) | 0% |
|  | 18mg+18mg Reteplase versus 0.9mg/kg alteplase | Mean age | ≥65 years | NA | NA | NA | NA |
|  |  |  | ＜65 years | 2 | 0.93 | 1.03(0.53,2.01) | 0% |
|  |  | Mean age | Caucasian | NA | NA | NA | NA |
|  |  |  | Asian | 2 | 0.93 | 1.03(0.53,2.01) | 0% |
| Death within 90 days | 0.25mg/kg Tenecteplase versus 0.9mg/kg alteplase | Mean age | ≥65 years | 7 | 0.71 | 0.96(0.78,1.19) | 0% |
|  |  |  | ＜65 years | 1 | 0.09 | 0.16(0.02,1.35) | NA |
|  |  | Race | Caucasian | 6 | 0.29 | 0.88(0.69,1.12) | 0% |
|  |  |  | Asian | 2 | 0.62 | 0.60(0.08,4.53) | 73% |
|  | 18mg+18mg Reteplase versus 0.9mg/kg alteplase | Mean age | ≥65 years | NA | NA | NA | NA |
|  |  |  | ＜65 years | 2 | 0.43 | 1.22(0.74,2.01) | 0% |
|  |  | Race | Caucasian | NA | NA | NA | NA |
|  |  |  | Asian | 2 | 0.43 | 1.22(0.74,2.01) | 0% |
| Serious adverse event | 0.25mg/kg Tenecteplase versus 0.9mg/kg alteplase | Mean age | ≥65 years | 4 | 0.51 | 1.18(0.71,1.97) | 79% |
|  |  |  | ＜65 years | 1 | 0.11 | 0.45(0.17,1.21) | NA |
|  |  | Race | Caucasian | 3 | 0.58 | 1.3(0.51,3.3) | 86% |
|  |  |  | Asian | 2 | 0.59 | 0.80(0.35,1.81) | 64% |
|  | 18mg+18mg Reteplase versus 0.9mg/kg alteplase | Mean age | ≥65 years | NA | NA | NA | NA |
|  |  |  | ＜65 years | 2 | 0.06 | 1.33(0.99,1.78) | 0% |
|  |  | Race | Caucasian | NA | NA | NA | NA |
|  |  |  | Asian | 2 | 0.06 | 1.33(0.99,1.78) | 0% |

**Table S11 The influence of the average baseline NIHSS score on the efficacy and safety of thrombolytic drugs**

| **Outcomes** | **Comparisons*** | **Subgroups(Average NIHSS)** | **Number of  comparisons** | **P value** | **Odds ratio（95%Cl）** | **I^2^** |
| --- | --- | --- | --- | --- | --- | --- |
| Excellent functional outcome at 90 days | Tenecteplase versus alteplase | ＜8 | 3 | 0.2 | 1.11 (0.95, 1.29) | 0 |
|  |  | 8-15 | 6 | 077 | 1.03(0.86,1.22) | 50% |
|  |  | ＞15 | 1 | 0.15 | 1.43(0.82,1.49) | NA |
|  | Reteplase versus alteplase | ＜8 | 2 | 0.0002 | 1.55(1.23,1.95) | 34% |
| Good functional outcome at 90 days | Tenecteplase versus alteplase | ＜8 | 3 | 0.58 | 0.95(0.8,1.13) | 0% |
|  |  | 8-15 | 6 | 0.78 | 0.78(0.46,1.31) | 72% |
|  |  | ＞15 | 1 | 0.06 | 1.77(0.97,2.99) | NA |
|  | Reteplase versus alteplase | ＜8 | 2 | 0.66 | 1.15(0.61,2.19) | 61% |
| Symptomatic intracranial haemorrhage | Tenecteplase versus alteplase | ＜8 | 3 | 0.56 | 1.16(0.7,1.91) | 0% |
|  |  | 8-15 | 5 | 0.74 | 1.08(0.68,1.72) | 22% |
|  |  | ＞15 | 1 | 1 | 1.00(0.06,16.21) | NA |
|  | Reteplase versus alteplase | ＜8 | 2 | 0.82 | 1.08(0.56,2.07) | 0% |
| Death within 90 days | Tenecteplase versus alteplase | ＜8 | 3 | 0.4 | 1.15(0.83,1.6) | 0% |
|  |  | 8-15 | 4 | 0.11 | 0.51(0.22,1.16) | 56% |
|  |  | ＞15 | 1 | 0.11 | 0.51(0.22,1.16) | NA |
|  | Reteplase versus alteplase | ＜8 | 2 | 0.53 | 1.17(0.71,1.91) | 0% |
| Serious adverse event | Tenecteplase versus alteplase | ＜8 | 3 | 0.88 | 1.01(0.84,1.23) | 0% |
|  |  | 8-15 | 1 | 0.82 | 1.04(0.74,1.46) | NA |
|  |  | ＞15 | 1 | 0.09 | 0.55(0.28,1.1) | NA |
|  | Reteplase versus alteplase | ＜8 | 2 | 0.07 | 1.31(0.98,1.75) | 0% |


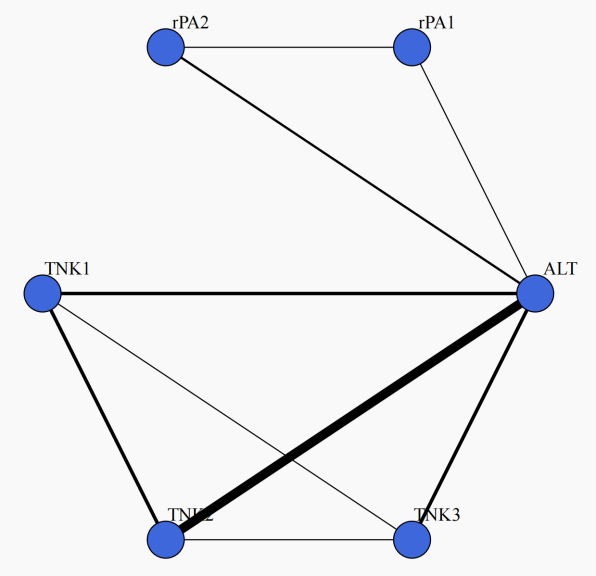

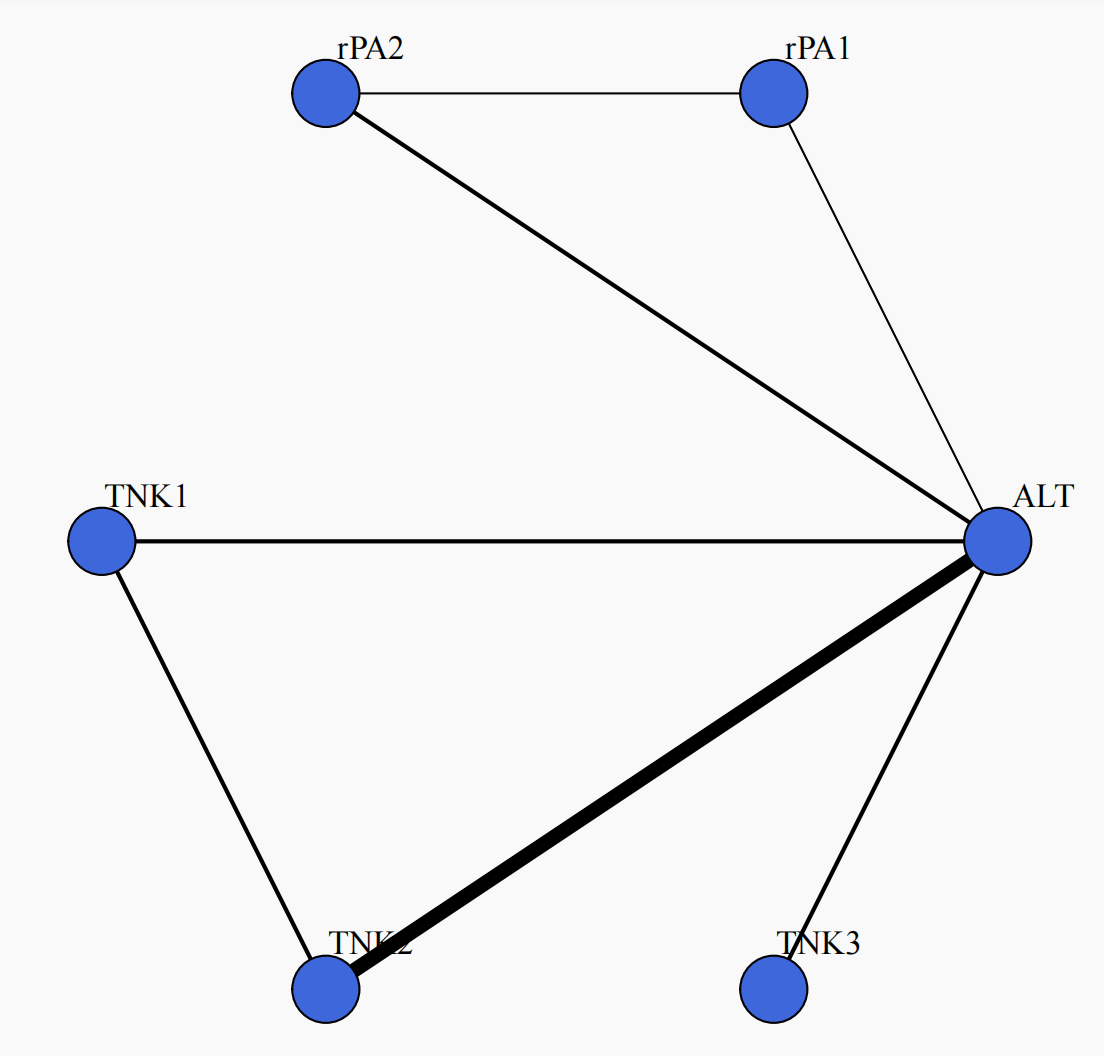


（A） （B）


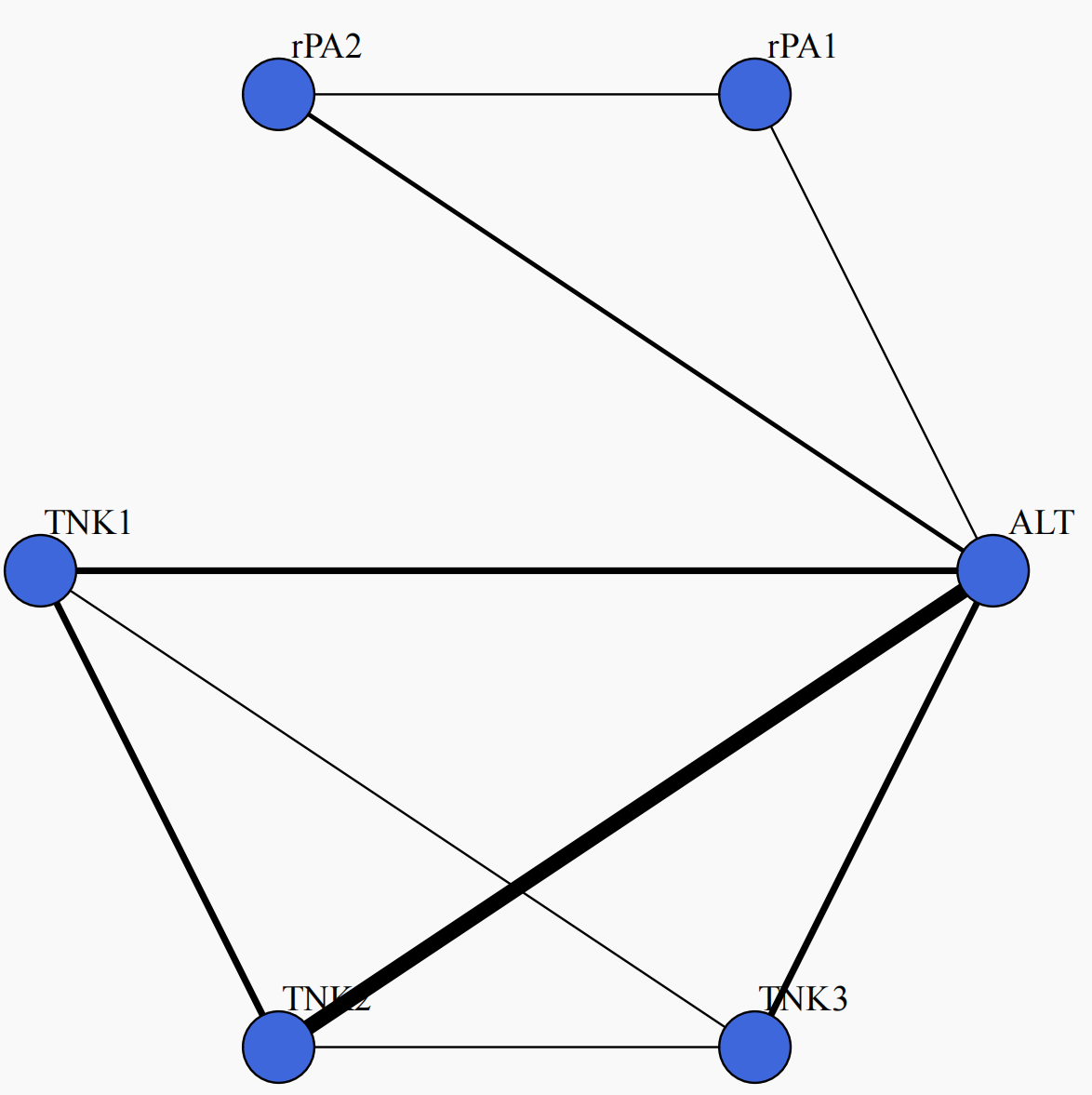

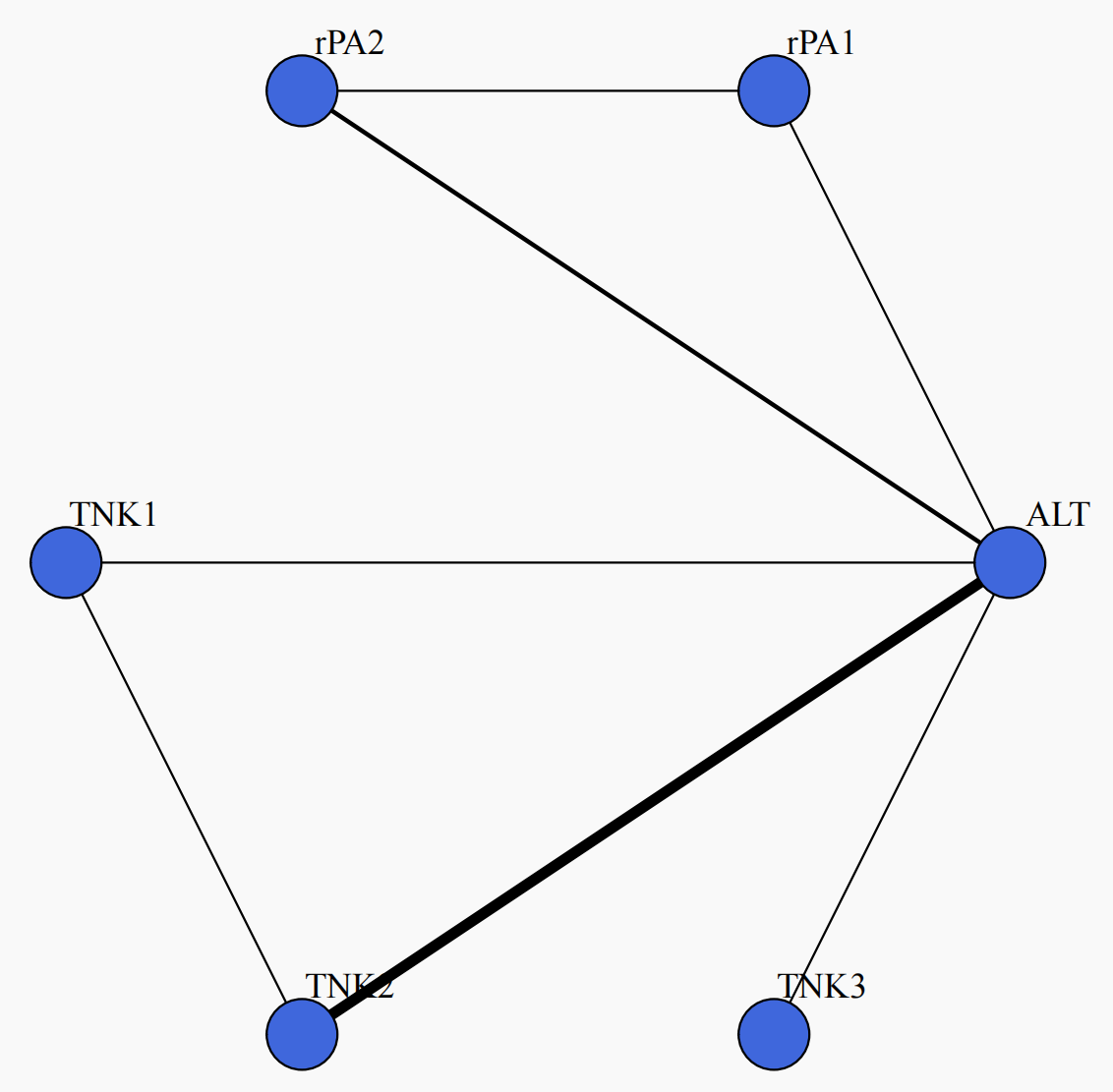


（C） （D）

**Figure S1 Network plot of studies for different outcomes.** Figure S1.A Network plot of studies for the excellent/death functional outcome at 90 days(including 29 arms). Figure S1.B Network plot of studies for the good functional outcome at 90 days(including 25 arms). Figure S1.C Network plot of studies for the sICH(including 27 arms). Figure S1.D Network plot of studies for the SAE(including 18 arms).


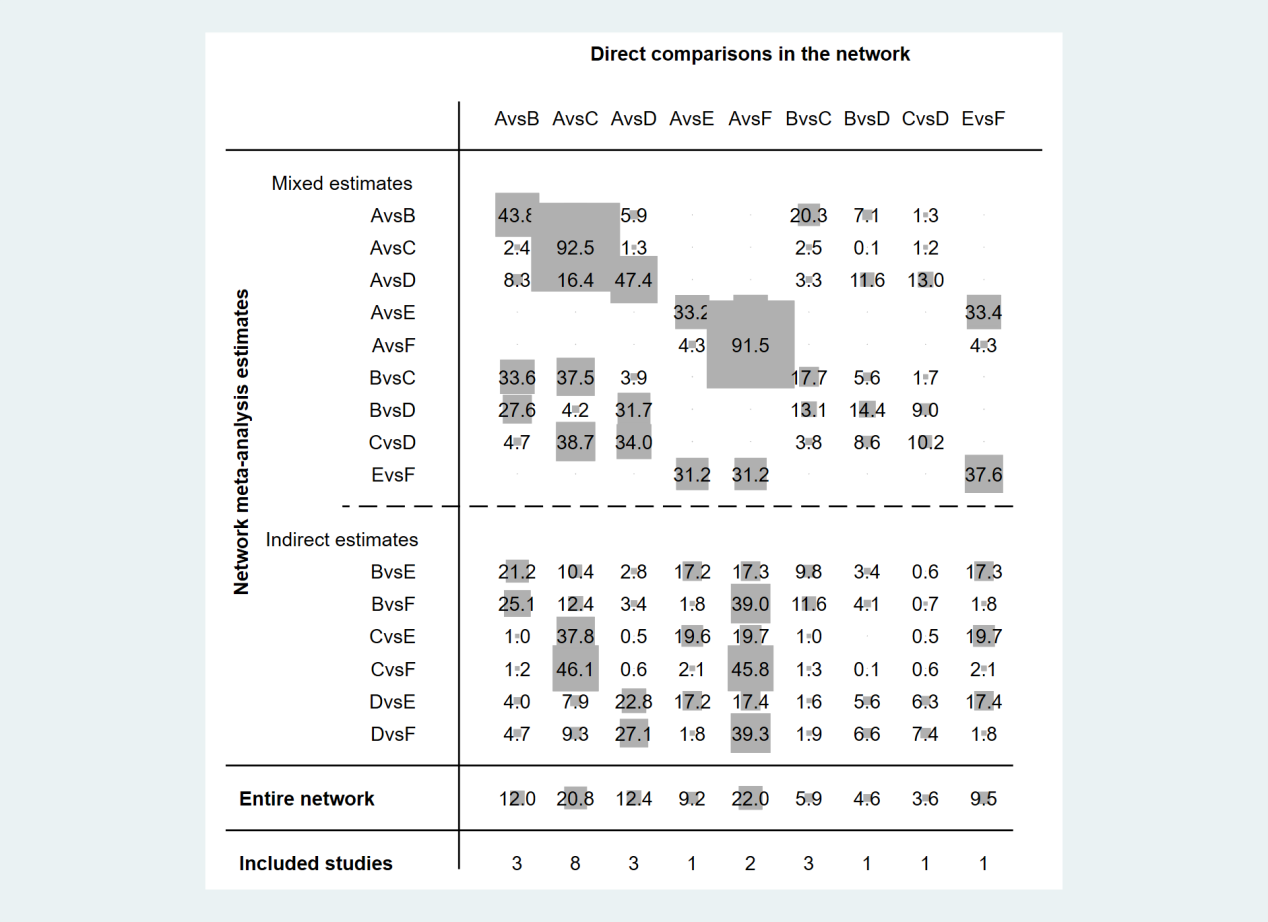


**Figure S2 The contribution to the network estimates.**


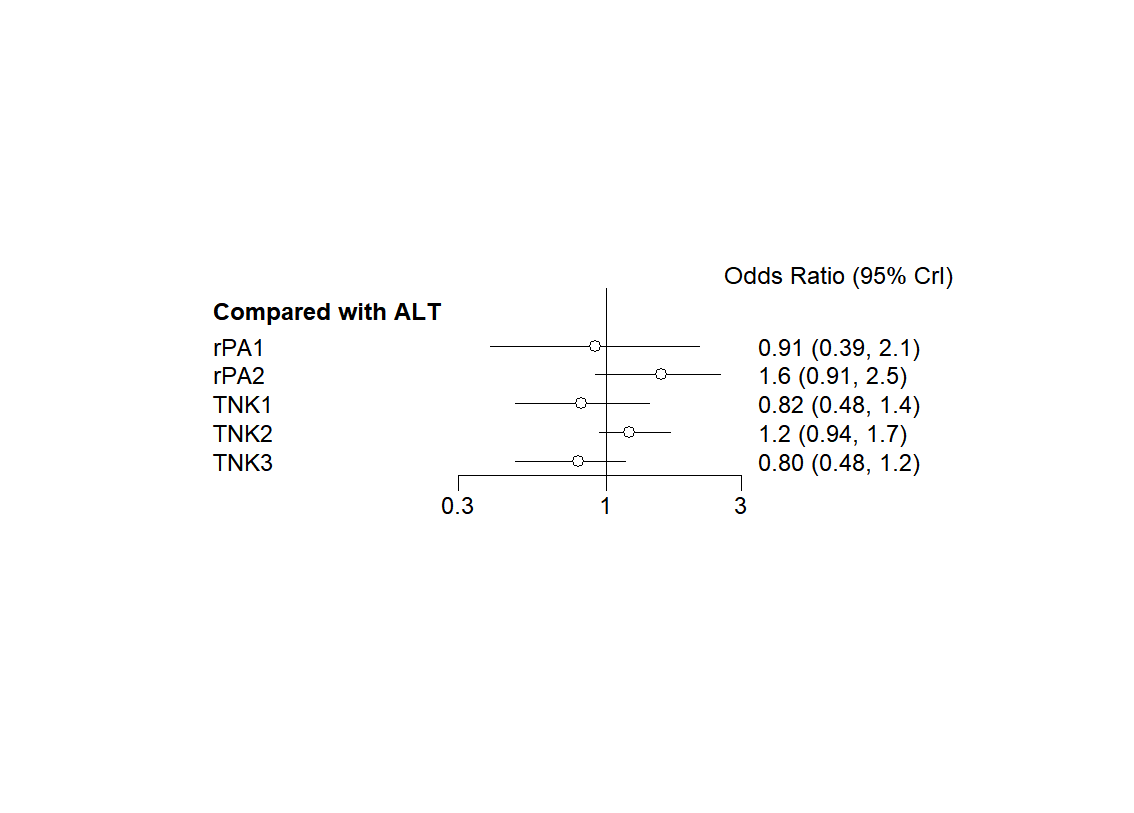


**Figure S3 The forest of different dose of the tenecteplase and reteplase on excellent functional outcome at 90 days.**


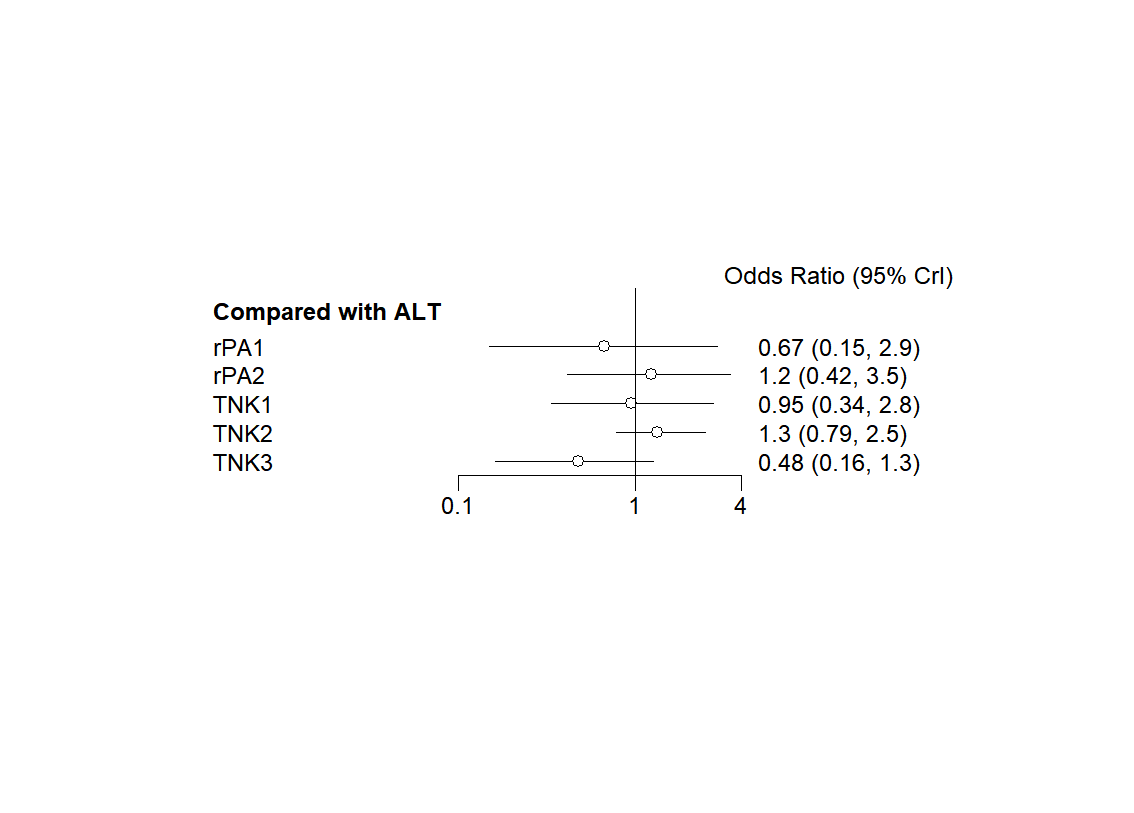


**Figure S4 The forest of different dose of the tenecteplase and reteplase on good functional outcome at 90 days.**


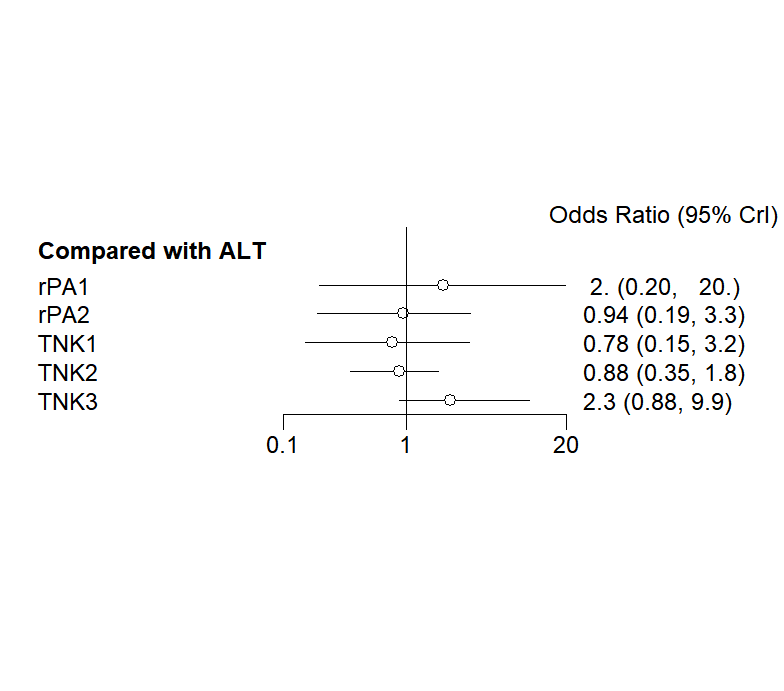


**Figure S5 The forest of different dose of the tenecteplase and reteplase on symptomatic intracranial haemorrhage.**


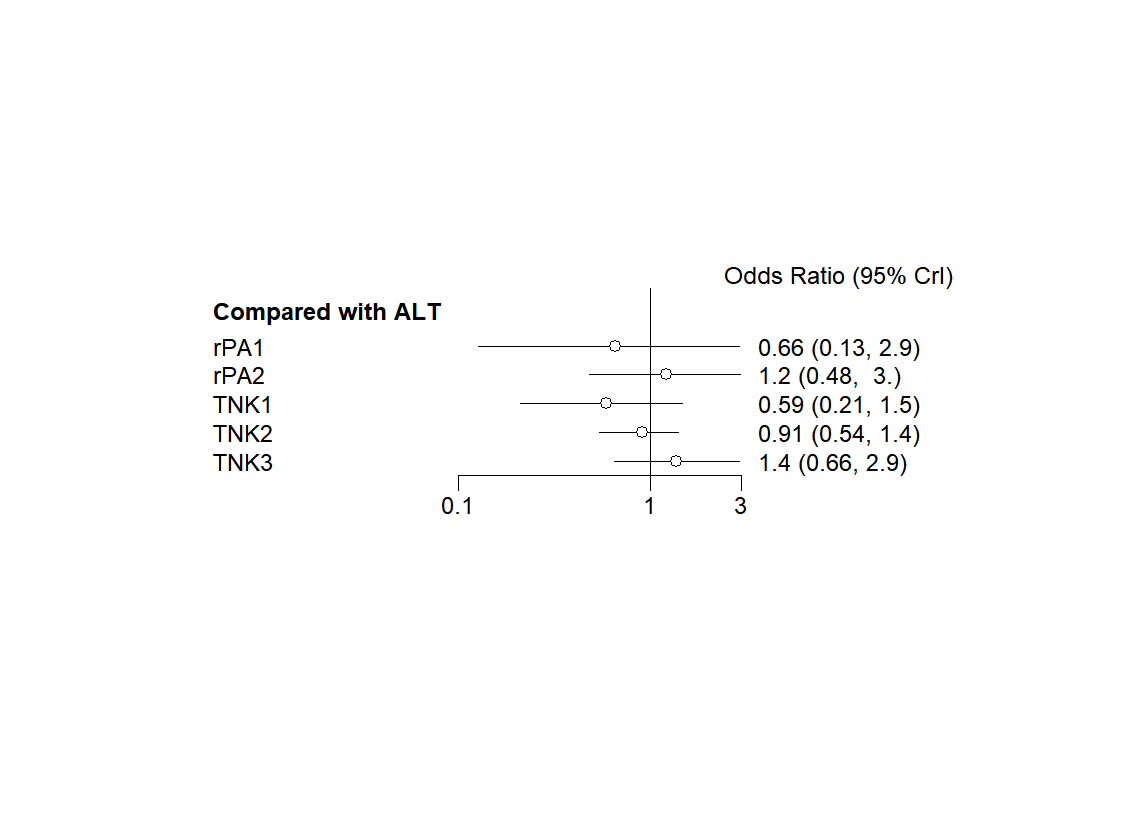


**Figure S6 The forest of different dose of the tenecteplase and reteplase on death within 90 days.**


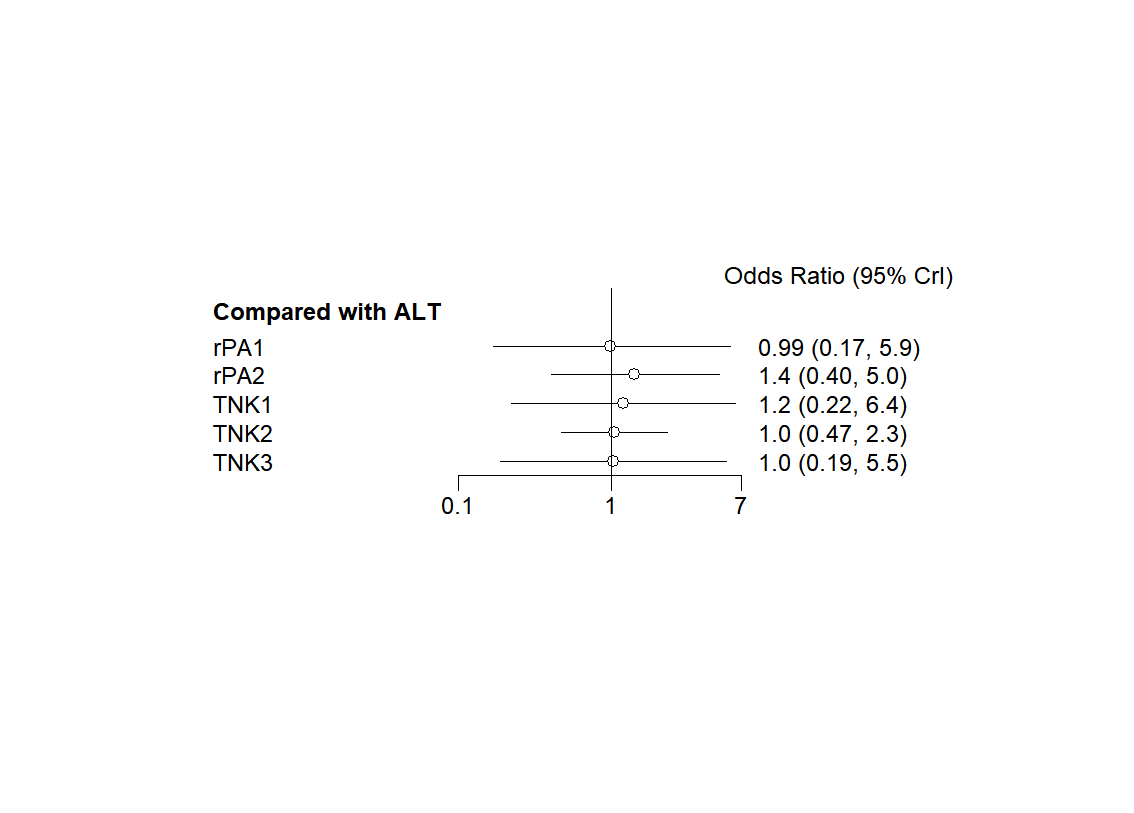


**Figure S7 The forest of different dose of the tenecteplase and reteplase on serious adverse event.**


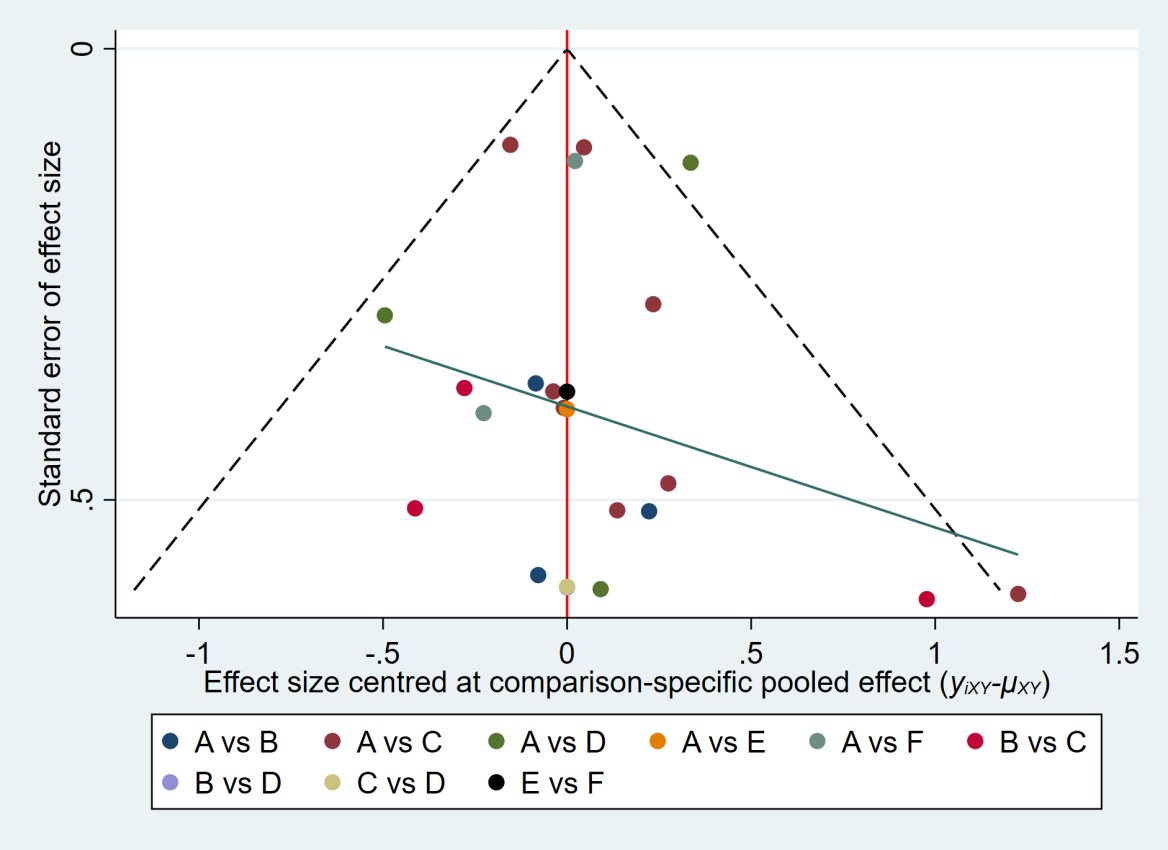


**Figure S8 Funnel plot for different dose of the tenecteplase and reteplase on excellent functional outcome at 90 days.**


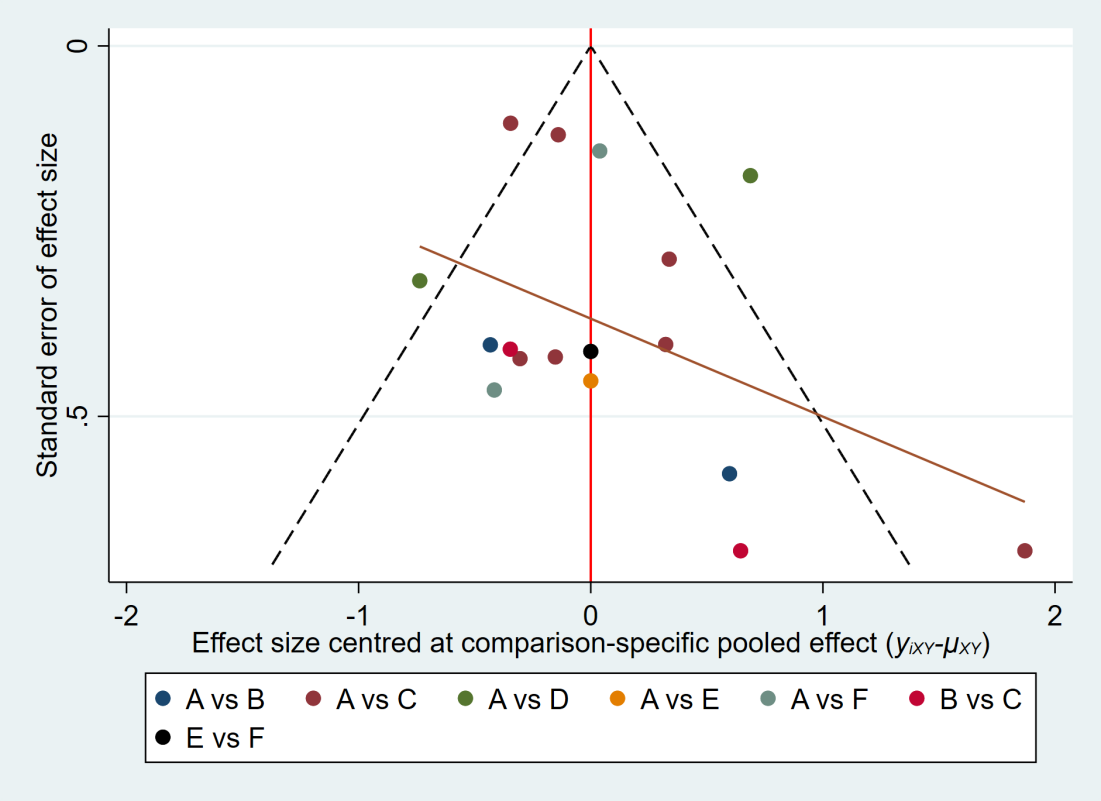


**Figure S9 Funnel plot for different dose of the tenecteplase and reteplase on good functional outcome at 90 days.**


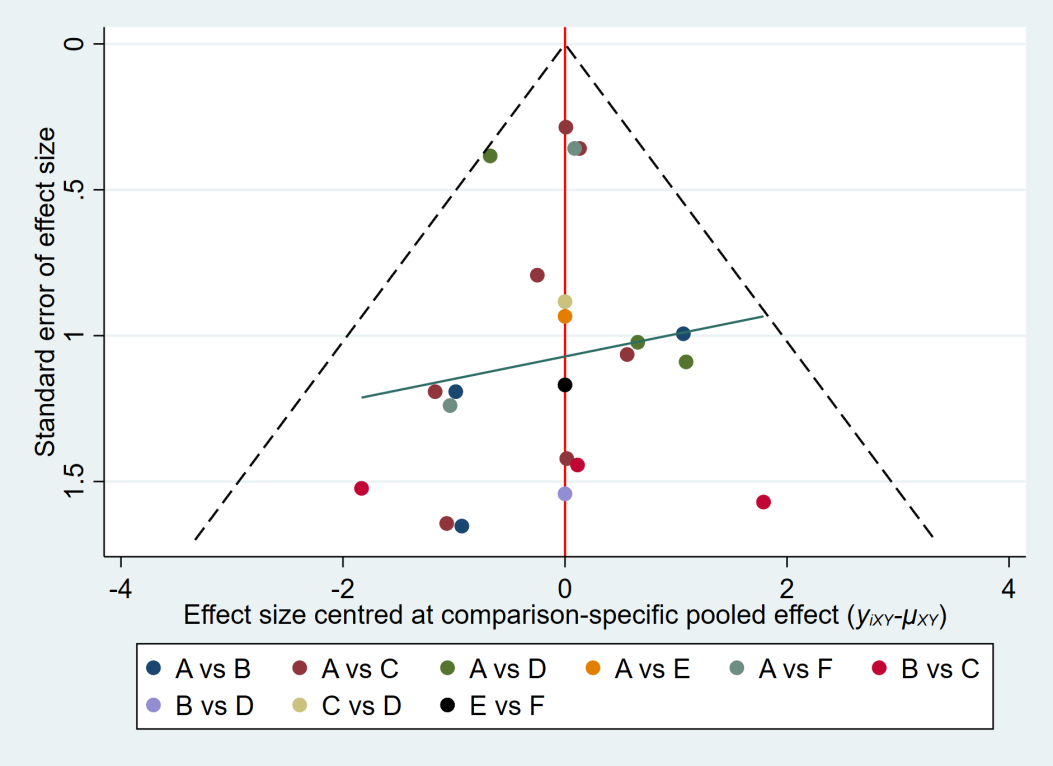


**Figure S10 Funnel plot for different dose of the tenecteplase and reteplase on symptomatic intracranial haemorrhage.**


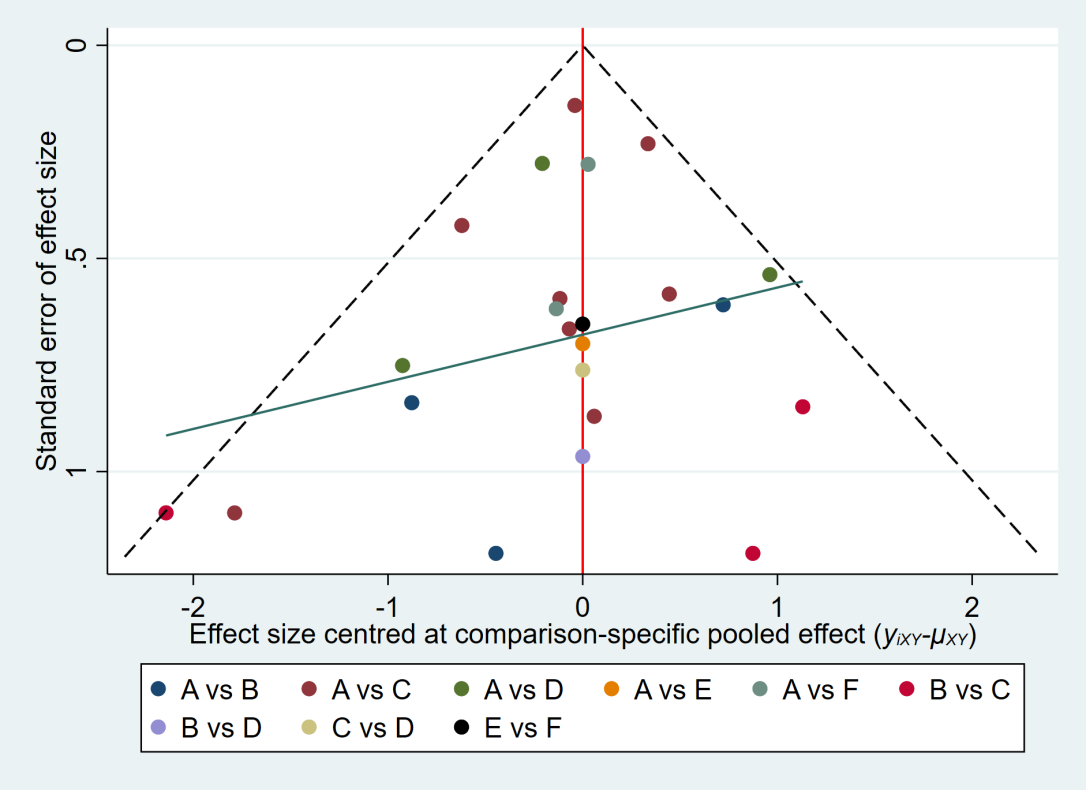


**Figure S11 Funnel plot for different dose of the tenecteplase and reteplase on death within 90 days.**


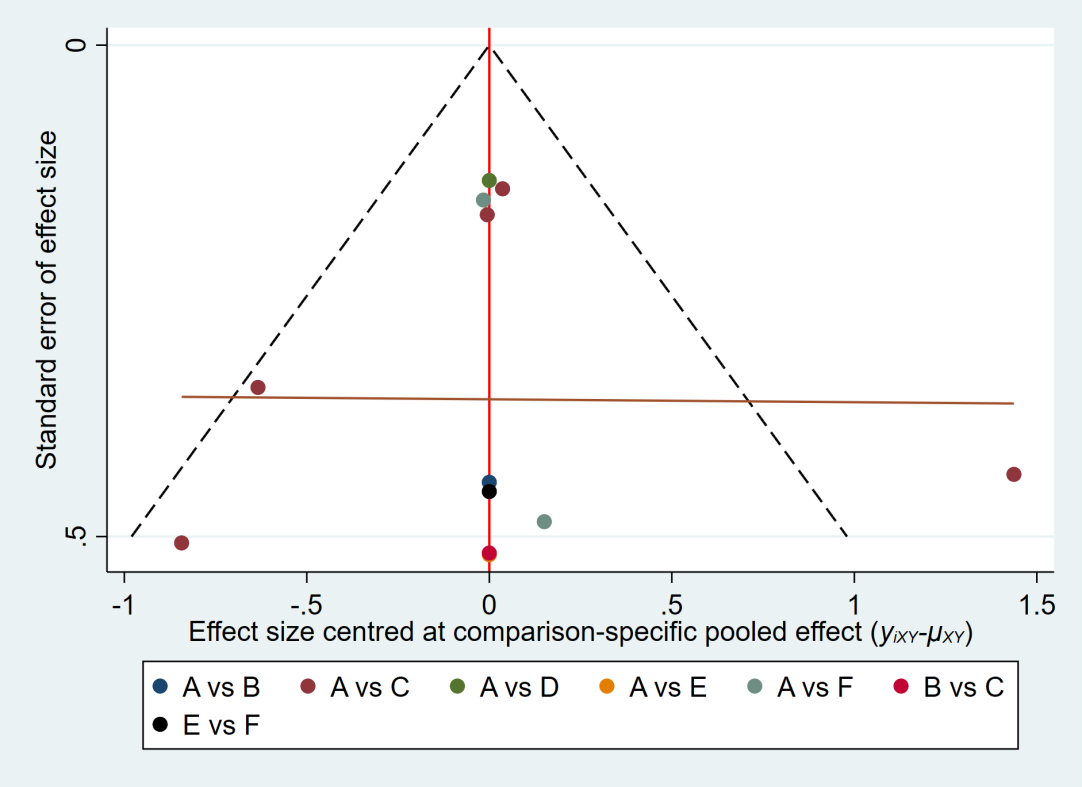


**Figure S12 Funnel plot for different dose of the tenecteplase and reteplase on serious adverse events.**
